# Development of a breast cancer invasion score to predict tumor aggressiveness and prognosis *via* PI3K/AKT/mTOR pathway analysis

**DOI:** 10.1038/s41420-025-02422-y

**Published:** 2025-04-09

**Authors:** Xiujuan Li, Ya Zhang, Jianping Gong, Wenjia Liu, Hanchen Zhao, Wei Xue, Zhaojun Ren, Jun Bao, Ziao Lin

**Affiliations:** 1 Department of General Surgery, Jiangsu Cancer Hospital, Jiangsu Institute of Cancer Research, The Affiliated Cancer Hospital of Nanjing Medical University, Nanjing, 210000 China; 2 Shanghai Key Laboratory of Maternal and Fetal Medicine, Clinical and Translational Research Center of Shanghai First Maternity and Infant Hospital, Frontier Science Center for Stem Cell Research, School of Life Sciences and Technology, Tongji University, Shanghai, 200092 China; 3 OmixScience Research Institute, OmixScience Co., Ltd., Hangzhou, 311199 China; 4 The Second Affiliated Hospital, Nanjing Medical University, Nanjing, 210011 China; 5 Department of Pathology, Jiangsu Cancer Hospital, Jiangsu Institute of Cancer Research, Nanjing Medical University Affiliated Cancer Hospital, Nanjing, 210000 China; 6 Department of Medical Oncology, Jiangsu Cancer Hospital, Jiangsu Institute of Cancer Research, The Affiliated Cancer Hospital of Nanjing Medical University, Nanjing, 210000 China; 7 Liangzhu Laboratory, Zhejiang University, Hangzhou, 311100 China

**Keywords:** Breast cancer, Prognostic markers

## Abstract

Invasiveness is a key indicator of tumor malignancy and is often linked to poor prognosis in breast cancer (BC). To explore the diverse characteristics of invasive cells, single-cell RNA sequencing (scRNA-seq) data from three ductal carcinoma stages were analyzed, classifying samples into invasion and non-invasion groups. Nine genes (*MCTS1*, *PGK1*, *PCMT1*, *C8orf76*, *TMEM242*, *QPRT*, *SLC16A2*, *AFG1L*, and *SPINK8*) were identified as key discriminators between these groups. A breast cancer invasion score (BCIS) model was developed using LASSO Cox regression, revealed that high BCIS correlated with poorer overall survival in TCGA-BRCA patients and was validated across GSE20685 and METABRIC datasets (five-year and ten-year survival). Functional experiments demonstrated that knockdown of *PGK1* or *PCMT1* inhibited tumor cell proliferation and reduced the phosphorylation levels of mTORC, P70S6K, S6, and AKT, indicating suppression of the PI3K/AKT/mTOR pathways. High-BCIS tumors exhibited enrichment in protein secretion and PI3K/AKT/mTOR pathways, associated with aggressiveness and therapy resistance. This study introduced the BCIS score, distinguishing invasion from non-invasion cells, linked to PI3K/AKT/mTOR pathways, offering insights into BRCA prognosis and tumor aggressiveness.

## Introduction

Breast cancer (BC) remains the leading cause of cancer-related deaths among women [1]. Data collected by the World Health Organization (WHO) and recent studies from 2021 and 2022 indicate that over 2.2 million women worldwide are diagnosed with BC, with more than 500,000 deaths, accounting for approximately 15–16% of cancer deaths and 25–30% of cancer cases [2–4]. Ductal carcinoma is the most common subtype of BC and has the potential to progress from in-situ carcinoma to invasive carcinoma [5]. Ductal carcinoma in situ (DCIS) is defined as the malignant proliferation of cells confined within the breast ducts and is considered a precursor to invasive BC, although most DCIS lesions are harmless [6–8]. However, if left untreated, about half of DCIS cases may progress to invasive ductal carcinoma (IDC), with DCIS cells penetrating the ductal basement membrane and invading the surrounding stroma [7, 9]. Recent studies have shown that DCIS coexisting with adjacent IDC exhibits very similar gene expression and copy number profiles, indicating a common origin for the progression [8]. IDC accompanied by lymph metastasis often presents more complex treatment demands and poorer clinical outcomes, representing a higher likelihood of further spread to distant organs, thereby reducing patient survival rates [10]. In-depth research into the molecular mechanisms underlying the transition from non-invasive to invasive BC can help understand the process of malignant transformation in BC and provide clues for identifying new therapeutic targets.

However, traditional prognostic biomarkers have long been ineffective in the early diagnosis of BC patients and cannot provide clues for cancer deterioration [11, 12]. For DCIS, treatment typically includes surgery and radiotherapy, but some DCIS cases may not progress to IDC [13]. There is currently a lack of effective biomarkers to identify low-risk patients, often leading to overtreatment. Additionally, current treatment strategies are usually based on clinical and pathological features. However, tumor heterogeneity in ductal carcinoma patients is high, and different patients may respond significantly differently to the same treatment [14]. While existing biomarkers have enabled personalized treatment to some extent, they remain inadequate in addressing complex tumor heterogeneity, particularly in predicting and managing treatment resistance. Furthermore, the prognostic value of biomarkers may vary depending on individual differences and tumor subtypes.

Previous studies have found abnormal methylation of *PAX6*, *BRCA2*, *PAX5*, *WT1*, *CDH13*, and *MSH6* in more than 50% of DCIS and adjacent IDC lesions, but the methylation of these genes showed no significant difference between DCIS and IDC [15]. It has not been possible to identify key biomarkers that specifically indicate the progression from DCIS to IDC, and there is still a need to explore other types of molecular or genetic changes to explain this progression. Similarly, while the prognostic and predictive significance of human epidermal growth factor receptor 2 (*HER2*) in invasive BC has been well established, *HER2* amplification is more associated with the DCIS stage but is not a key factor in the transition from DCIS to IDC [16]. These biomarkers have shown potential in previous studies, but no single marker has been widely accepted as the key predictor for the transition of ductal carcinoma from localized to invasive, and then to metastatic stages. Therefore, current research continues to explore combinations of multiple molecular mechanisms and biomarkers to more accurately predict this cancer progression. Through multi-biomarker models and larger-scale studies, we can better understand the progression mechanisms of ductal carcinoma and develop more effective clinical prediction tools.

The PI3K-AKT-mTOR signaling pathway plays a crucial role in the initiation and progression of tumorigenesis, including breast cancer, by regulating essential cellular functions such as survival, proliferation, metabolism, and angiogenesis. Dysregulation of this pathway leads to enhanced tumor cell growth, evasion of cell death, and increased metastatic potential. In breast cancer, aberrant activation of the PI3K-AKT-mTOR axis has been linked to resistance to therapies, poor prognosis, and the transition from early-stage lesions like DCIS to invasive carcinoma, highlighting its significance as both a driver of tumorigenesis and a potential therapeutic target [17–19].

Single-cell RNA sequencing (scRNA-seq) reveals the state and function of each cell by isolating individual cells, capturing their transcriptome, and generating sequencing libraries at the single-cell level [20]. This technology allows for an in-depth analysis of the progression from DCIS to IDC and IDC_LM, by revealing cell heterogeneity, analyzing the diversity of cell types, and identifying the dynamic changes of key genes and pathways, thus helping to understand the mechanisms of cancer metastasis and promoting the development of personalized treatment. In this study, we conducted a comprehensive analysis of scRNA-seq data from ductal carcinoma to deeply understand the molecular complexity of ductal carcinoma cells and to identify unique marker genes associated with the three stages of ductal carcinoma. Additionally, the study integrated multi-dataset analysis to develop a breast cancer invasion score (BCIS) model, which aims to be a powerful prognostic tool for ductal carcinoma. We validated its accuracy based on an independent cohort GSE20685 from the Gene Expression Omnibus (GEO) database and five years and ten years survival data from the TCGA-BRCA cohort. Moreover, we conducted an in-depth study of the interaction between BCIS and the immunosuppressive tumor microenvironment (TME) and performed a potential functional analysis in the context of BRCA. Finally, through experimental validation, we determined whether marker genes play a key role in the progression of ductal carcinoma to evaluate their feasibility as potential therapeutic targets or prognostic markers.

## Results

### Cellular Atlas of Ductal Carcinoma

**Fig. 1:**
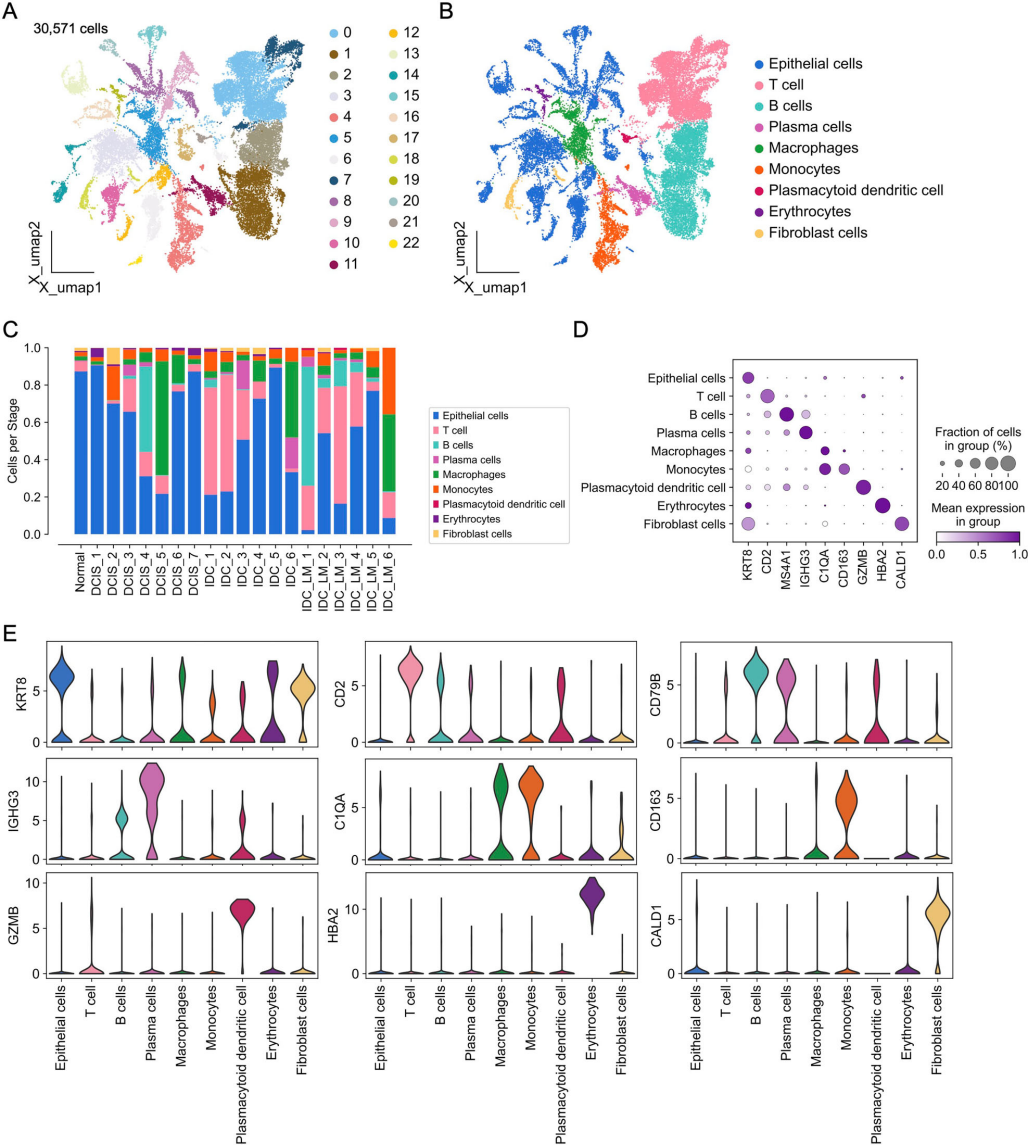
Analysis of the breast cancer single-cell atlas. **A** Uniform manifold approximation and projection (UMAP) visualization of 30,577 cells categorized into 22 distinct clusters. **B** UMAP delineation of nine principal cell lineages (Epithelial cells, T cells, B cells, Plasma cells, Macrophages, Monocytes, Plasmacytoid dendritic cells, Erythrocytes, and Fibroblast cells) derived from BC patient samples. **C** Bar plot showing the distribution of cell types across Normal, DCIS, IDC, and IDC-LM stages. **D** Dot plot illustrating the expression of selected canonical marker genes across each cell type. **E** Violin plot for expression of three canonical marker genes (*KRT8*, *IGHG3*, *GZMB*, *CD2*, *C1QA*, *HBA2*, *CD79B*, *CD163*, *CALD1*) in each cell type.

This study performed a comprehensive analysis of the scRNA-seq dataset GSE195861 and constructed a cellular atlas based on it. The dataset contains 20 tissue samples, including 1 from Normal sample, 7 from patients diagnosed with DCIS, 6 from IDC patients, and 6 from IDC_LM samples (Fig. S1A). Additionally, we obtained one normal breast tissue sample from a DCIS patient who underwent a mastectomy to exclude the mixing of normal cells in DCIS and IDC samples. After quality control and data preprocessing, “n_pcs=50” was set for principal component analysis (PCA). Construct a neighborhood graph to highlight the most variable genes, and set n_neighbors to 15. Using the Leiden algorithm with Scanpy [21], we identified 23 distinct cell clusters from a total of 30,571 cells (Fig. 1A). Furthermore, we annotated these 23 cell clusters using pySCSA [22, 23] and canonical cell markers, ultimately classifying them into nine primary cell subsets, including epithelial cells, T cells, B cells, plasma cells, macrophages, monocytes, plasmacytoid dendritic cells, erythrocytes, and fibroblast cells (Figs. 1B, C and S1B). As DCIS progresses to IDC and subsequently to IDC_LM, the composition of the tumor microenvironment undergoes significant changes. Notably, the involvement of immune cells gradually increases, indicating that the immune response becomes more active during the cancer progression. Additionally, the expression of 27 canonical marker genes in each cell subsets was described (Fig. S1C, D). These cell subsets exhibited distinct characteristics through high expression of *KRT8*, *CD2*, *MS4A1*, *IGHG3*, *C1QA*, *CD163*, *GZMB*, *HBA2*, and *CALD1* (Figs. 1D, E and S1C). In summary, we describe the overall cellular atlas of 20 samples as a basis for further analysis.

### Characterization of different epithelial cell clusters

**Fig. 2:**
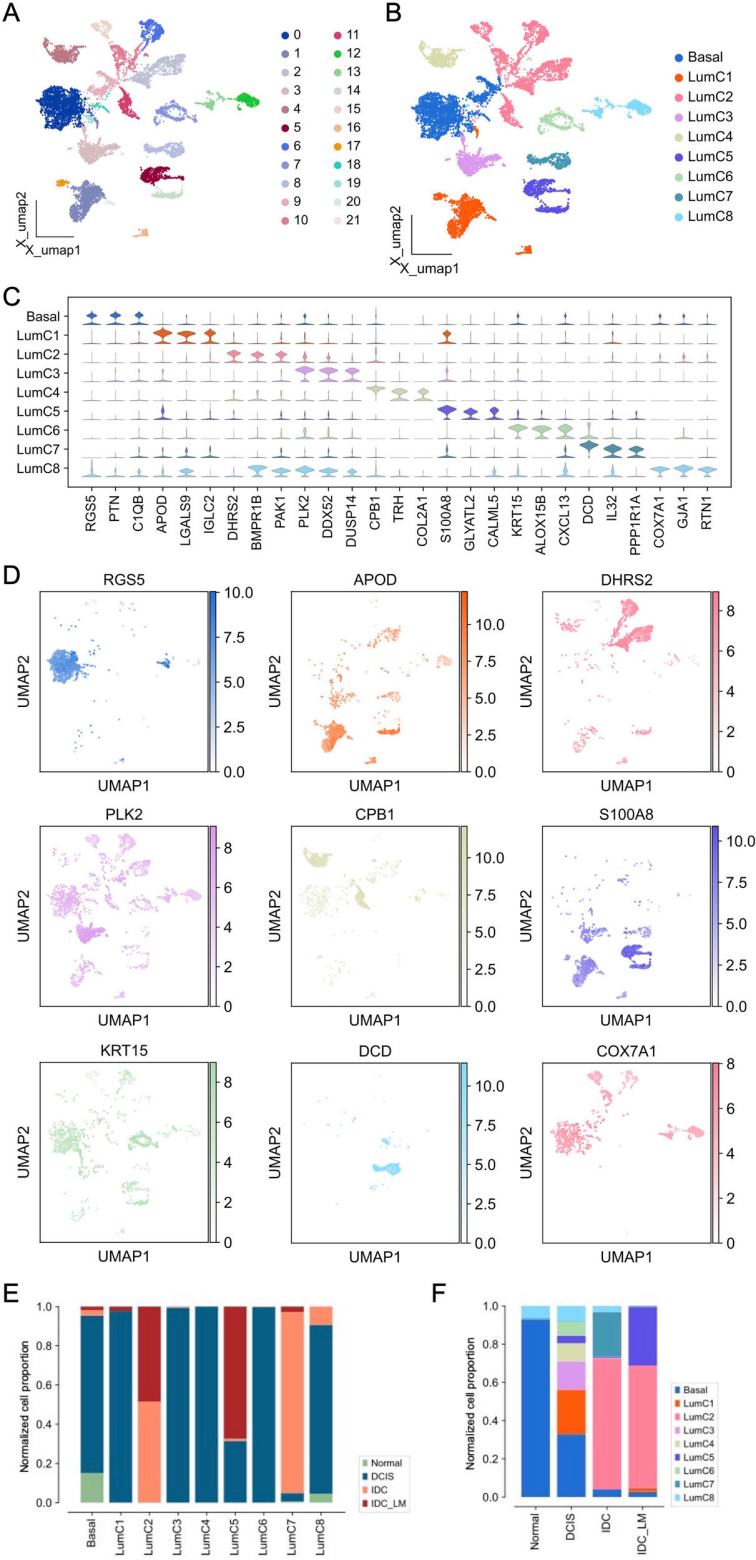
Characterization of epithelial cell heterogeneity in BC. **A** UMAP visualization of epithelial cells categorized into 22 distinct clusters. **B** UMAP plot of epithelial cells, colored by nine luminal subtypes. **C** Violin plot of differential gene expression across luminal subtypes in BC. **D** Umap plot of the expression of nine canonical marker genes in epithelial cells. **E** The proportion of different stages of BC development in different luminal subtypes. **F** The proportion of luminal subtypes in different BC development stages.

Epithelial cells are the primary origin cells of BC, and their mutations and variations are crucial for the progression of BC from DCIS to IDC. To study the heterogeneity of luminal epithelial cells in depth, we re-clustered epithelial cells from 20 samples (Fig. S2A). The UMAP plot of the epithelial cell lineage shows 22 clusters (Fig. 2A). Additionally, the distribution and aggregation of cells at different stages indicate that Normal, DCIS, IDC, and IDC_LM cells each have distinct clustering regions in the feature space, reflecting the unique gene expression characteristics of each cell type (Fig. S2B). Further differential analysis of the epithelial cell lineage successfully divided it into nine different epithelial cell subsets (Fig. 2B). These nine epithelial cell subgroups are characterized by the differential expression of genes such as *RGS5*, *PTN*, *C1QB*, *APOD*, *LGALS9*, *IGLC2*, *DHRS2*, *BMPR1B*, *PAK1*, *PLK2*, *DDX52*, *DUSP14*, *CPB1*, *TRH*, *COL2A1*, *S100A8*, *GLYATL2*, *CALML5*, *KRT15*, *ALOX15B*, *CXCL13*, *DCD*, *IL32*, *PPP1R1A*, *COX7A1*, *GJA1*, and *RTN1* (Fig. 2C). For example, the Basal subgroup is notably marked by the significant expression of *RGS5*, *PTN*, and *C1QB*. Additionally, we described the overall expression patterns of nine significantly expressed differential genes in epithelial cell subsets (Fig. 2D). Overall, most cell markers exhibit subgroup-specific expression patterns, some differential genes also exhibit specific expression characteristics at different stages of DCIS to IDC_LM (Fig. S2C). Furthermore, the proportion of cells within each epithelial cell subgroup suggests that different epithelial cell subgroups may play distinct roles in cancer progression and metastasis, with specific subgroups potentially being associated with particular types and stages of cancer (Fig. 2E). Notably, in Normal and DCIS cells, the Basal subgroup predominates. However, in IDC and IDC_LM, the proportion of Basal cells significantly decreases, suggesting that Basal cells may play a reduced role in cancer progression and metastasis (Figs. 2F and S2D). Conversely, the significant increase in the proportions of LumC2 and LumC5 subgroups in IDC and IDC_LM indicates that these subgroups may be associated with the invasiveness and metastatic potential of the cancer.

### Construction of prognostic model based on invasive and non-invasive cell feature genes

**Fig. 3:**
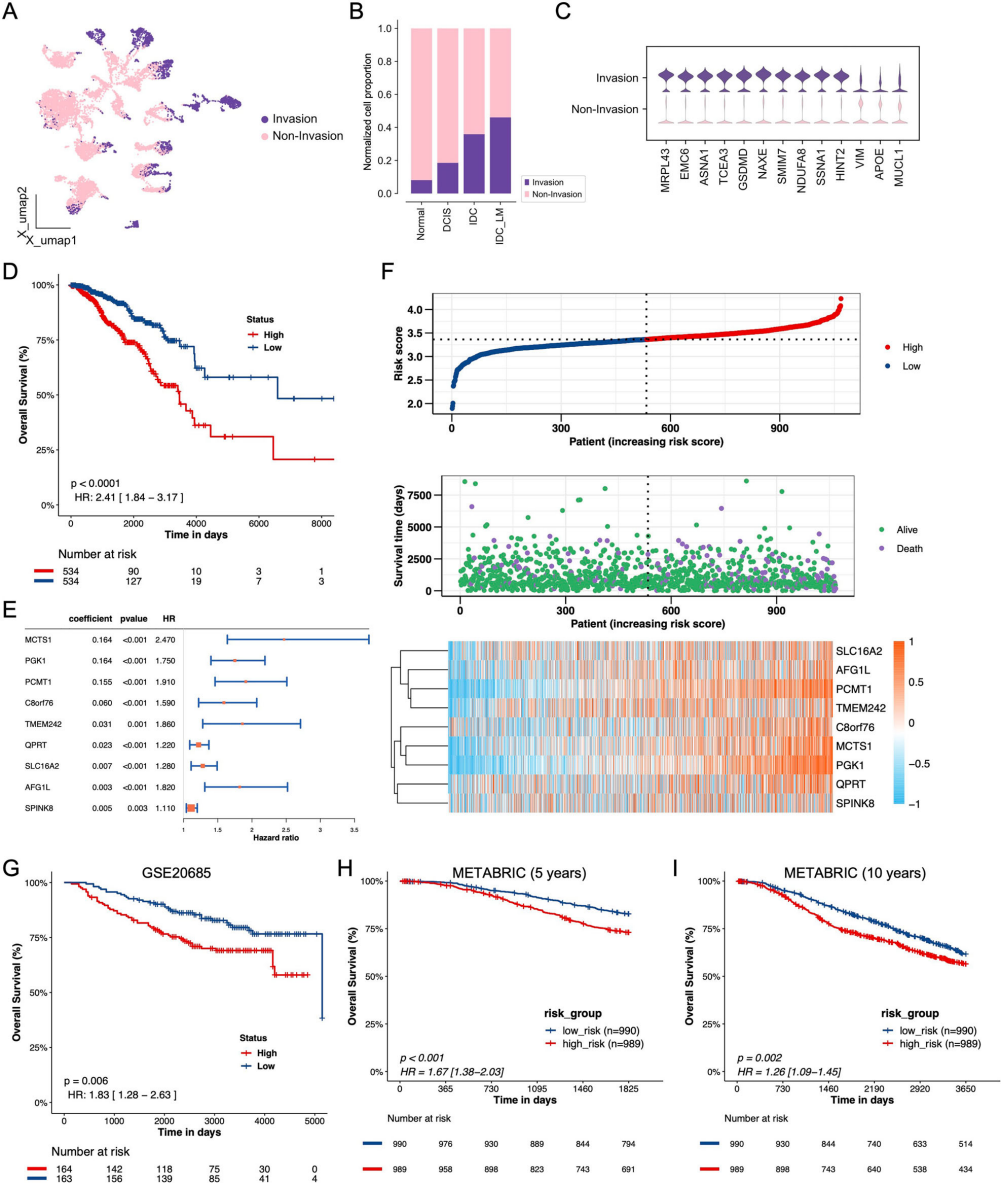
Characterization of invasive potential and prognostic value of breast ductal carcinoma cell subtypes. **A** UMAP plot of invasion and non-invasion cells, colored by two cell types. **B** Proportion of two cell types in different stages of ductal carcinoma development. **C** Violin map of thirteen representative expressed genes in invasive and non-invasive cell types. **D** Kaplan-Meier curves of survival analysis compared the overall survival of TCGA-BRCA patients between high-BCIS and low-BCIS groups. **E** Hazard ratios of nine signature genes in univariate cox models that were significantly associated with overall survival. **F** The distribution of risk score (top), survival status (middle), and expression (bottom) of the identified nine cell marker genes. Kaplan-Meier curves of survival analysis compared the overall survival of high-BCIS and low-BCIS groups in (**G**) GSE20685, (**H**) 5 years of survival data from METABRIC, (I) 10 years of survival data from METABRIC.

After calculating the CNV scores for Basal and luminal epithelial cells, we observed that all epithelial cells were categorized into invasive and non-invasive groups. Non-invasive cells are more widely dispersed, whereas invasive cells predominantly cluster in the IDC and IDC_LM regions (Fig. 3A). Furthermore, as the cancer progresses from Normal to DCIS, IDC, and eventually IDC_LM, the proportion of invasive epithelial cells steadily increases (Fig. 3B). This suggests that genomic instability and cellular invasiveness are critical drivers of BC progression and metastasis. Interestingly, the marker genes *MRPL43*, *EMC6*, *ASNA1*, *TCEA3*, *GSDMD*, *NAXE*, *SMIM7*, *NDUFA8*, *SSNA1*, and *HINT2* were highly expressed in the Invasive groups, while *VIM*, *APOE*, and *MUCL1* were highly expressed in the Non-Invasive groups (Fig. 3C and Figure S3A). Furthermore, the aforementioned marker genes of the Invasive groups were highly expressed in IDC and IDC_LM compared to DCIS samples. In contrast, the marker genes of the Non-Invasive groups were highly expressed in DCIS. This indicates that the Invasive groups are more aggressive (Fig. S3B-S3C).

We identified differentially expressed genes (DEGs) for each cluster by comparing the Invasive and Non-Invasive groups. To construct prognostic features from these DEGs, we conducted a Least Absolute Shrinkage and Selection Operator (LASSO) Cox proportional hazards regression analysis, using the TCGA-BRCA cohort as the training set. Ultimately, nine of the most predictive genes were selected for the BCIS prediction model (Fig. 3D). Risk score = (0.164 * *MCTS1* expression) + (0.164 * *PGK1* expression) + (0.155 * *PCMT1* expression) + (0.060 * *C8orf76* expression) + (0.031 * *TMEM242* expression) + (0.023 * *QPRT* expression) + (0.007 * *SLC16A2* expression) + (0.003 * *AFG1L* expression) + (0.005 * *SPINK8* expression) (Fig. 3E). We described the distribution of risk score, survival status, and gene expression level (Fig. 3F), finding that patients who died were mainly concentrated in the high-BCIS. We analyzed the expression of nine prognostic genes across different samples and found that *MCTS1*, *PGK1*, and *PCMT1* were significantly higher in IDC and IDC_LM compared to DCIS and Normal samples (Fig. S3D). Additionally, *PGK1* and *PCMT1* were more enriched in the high-BCIS group (Fig. 3F).

Further analysis was performed using the risk score of the GSE20685 dataset, which divided patients into high-BCIS (*n* = 164) and low-BCIS (*n* = 163). Kaplan-Meier (KM) analysis demonstrated that the overall survival (OS) of the high-risk group was significantly lower than that of the low-risk group (Fig. 3G, HR = 1.83, *p* = 0.006).

To further evaluate the accuracy of this prognostic risk model, patients in the TCGA-BRCA dataset were also divided into high-risk (*n* = 989) and low-risk (*n* = 990) groups and conducted KM analysis on five-year and ten-year survival data. The results consistently showed that in both five-year (Fig. 3H, HR = 1.67, *p* < 0.001) and ten-year (Fig. 3I, HR = 1.26, *p* = 0.002) survival data, the high-risk group had significantly worse prognosis than the low-risk group. These findings indicate that BCIS can serve as an effective tool for predicting patient prognosis.

We conducted a protein-protein interaction (PPI) analysis for 9 prognostic genes. The results reveal that several of these genes are interconnected, indicating potential interactions between their protein products (Fig. S3E). Notably, these 9 genes are central nodes in the network, suggesting they play a significant role in the protein interaction landscape. Given that some of these genes, *MCTS1*, *PGK1*, and *PCMT1* were previously identified as being highly expressed in the invasive groups, their prominent positions in this interaction network further support their involvement in cancer invasion. The interactions they participate in could be crucial for promoting invasive characteristics, such as cellular movement, survival in new environments, or evading immune responses. Additionally, *SPP1*, *CD24*, *LDHA*, and *ETFA* are known to promote tumor invasion and metastasis in various cancers.

### Validation of the BCIS in different independent cohorts

**Fig. 4:**
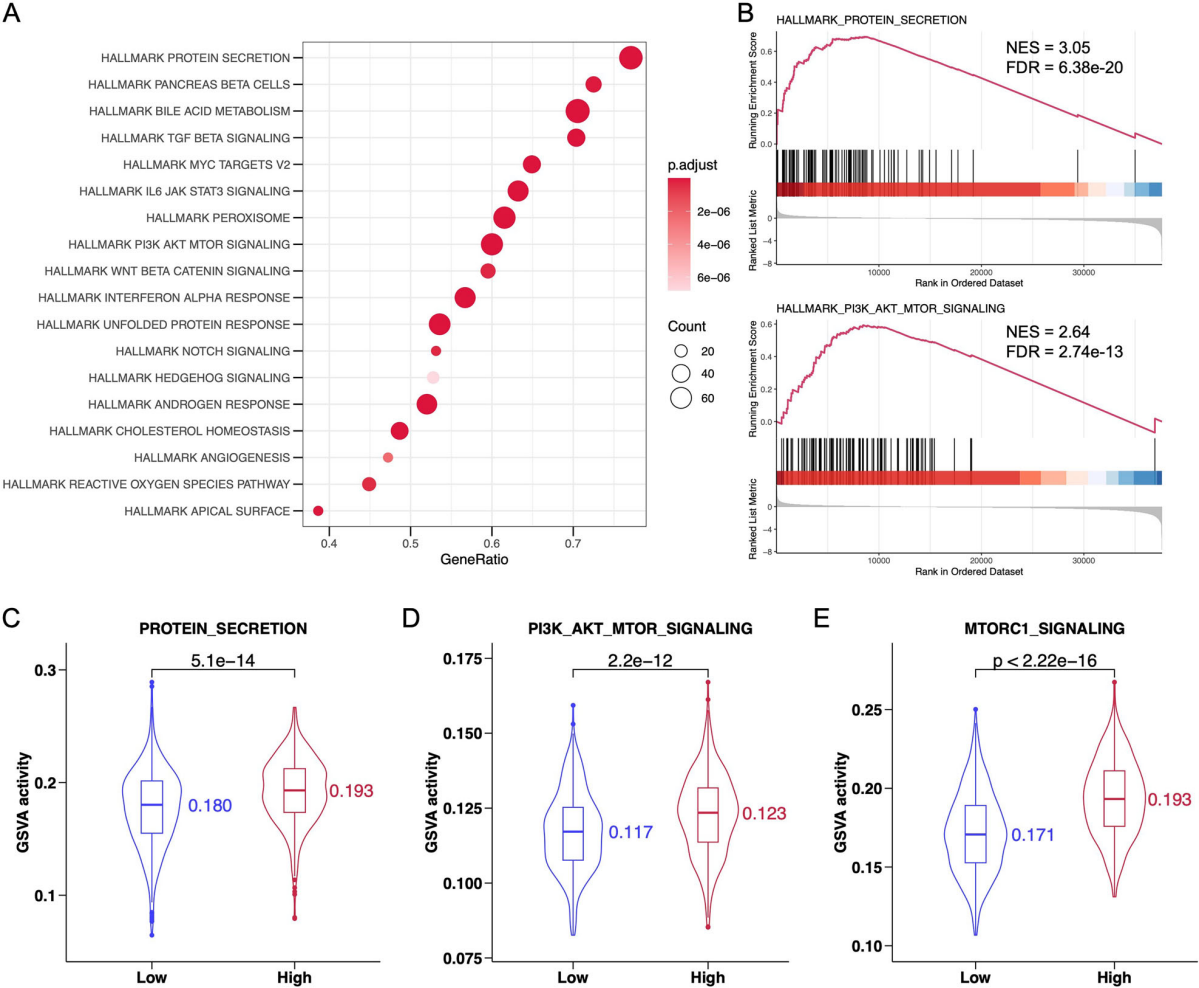
Function analysis between high-BCIS and low-BCIS groups. **A** Functional enrichment of high-BCIS and low-BCIS groups in TCGA-BRCA. **B** GSEA analysis of hallmark protein secretion signaling and hallmark PI3K/AKT/mTOR signaling pathway. GSVA activity analysis of (**C**) protein secretion, (**D**) PI3K/AKT/mTOR signaling, and (E) MTORC1 signaling pathway.

To understand the biological functions and mechanisms related to the risk scores, we performed Gene Set Enrichment Analysis (GSEA) with the primary goal of identifying pathways influenced by genes associated with the risk scores. Using the hallmark gene set (h.all.v2023.2.Hs.symbols.gmt) as a reference, we observed significant enrichment of hallmark protein secretion, hallmark bile acid metabolism and hallmark PI3K/AKT/mTOR signaling in the high-BCIS group (Fig. 4A). More detailed GSEA analysis showed that DEGs associated with higher risk scores were mainly related to hallmark protein secretion (Fig. 4B, NES = 3.05, FDR = 6.38e-20) and hallmark PI3K/AKT/mTOR signaling (NES = 2.64, FDR = 2.74e-13).

Using Gene Set Variation Analysis (GSVA), we further assessed the activity differences in biological pathways between different risk score groups. The results showed higher GSVA activity for protein secretion (Fig. 4C, *p* = 5.1e-14) and PI3K/AKT/mTOR signaling (*p* = 2.2e-12) in the high-BCIS group (Fig. 4D). Notably, the GSVA analysis also revealed significantly higher MTORC1 signaling GSVA activity in the high-BCIS group (Fig. 4E, *p* < 2.22e-16), indicating that the MTORC1 signaling might also be activated.

### Functional enrichment analysis of the BCIS related genes

**Fig. 5:**
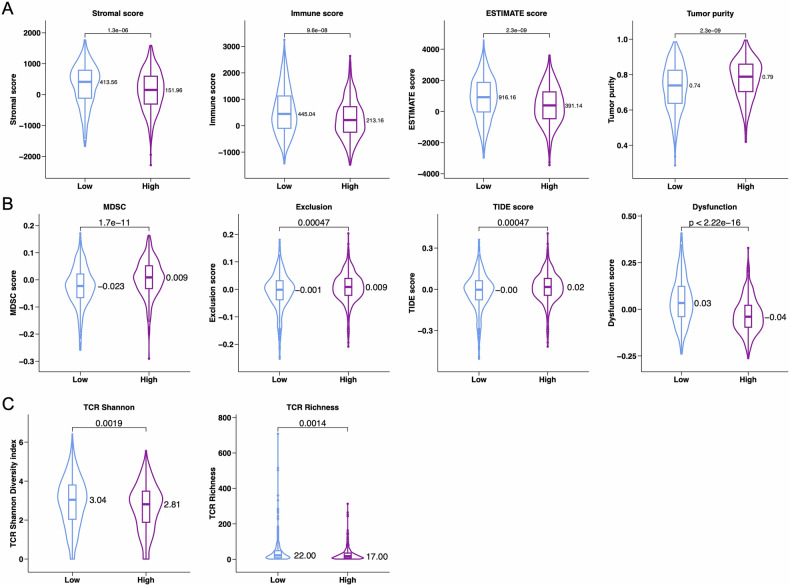
Tumor microenvironment analysis between high-BCIS and low-BCIS groups. TIDE analysis in high-BCIS and low-BCIS groups, including (**A**) Stromal score, Immune score, ESTIMATE score and Tumor purity (**B**) MDSC, Exclusion, TIDE score and Dysfunction. **C** The role of BCIS in predicting immunotherapeutic benefit. TCR Shannon and TCR Richness between high-BCIS and low-BCIS groups.

To gain a deeper understanding of the characteristics and roles of the risk score groups, we compared the differences between low-risk and high-risk groups based on multiple immune and tumor characteristic indices, including Stromal score, Immune score, ESTIMATE score, MDSC score, Exclusion score, TIDE score, Dysfunction score (Figs. 5A, S4A and S4B). The results showed that patients in the high-risk group had significantly higher scores in MDSC (Fig. 5B, *p* = 1.7e-11), Exclusion (*p* = 0.00047), and TIDE (*p* = 0.00047). These gene characteristic indices directly indicate that immune evasion is achieved through T-cell exclusion and dysfunction, suggesting a poorer prognosis and a less favorable response to immunotherapy.

### Immune and tumor characteristics differ by the BCIS

TCR analysis has become an important biomarker for assessing antitumor immune activity. High TCR shannon diversity index is the foundation for an adaptive immune system to effectively defend against various pathogens and tumors, while high TCR richness indicates a broad repertoire of T-cell receptors in an individual, enabling the recognition and response to a wider range of antigens, which is crucial for effective immune surveillance and defense. We evaluated TCR diversity using the TCR repertoire database and compared the differences between high-risk and low-risk groups. In this study, we found that the high-risk group had significantly lower TCR Shannon diversity index (*p* = 0.0019) and TCR richness (*p* = 0.0014) compared to the low-risk group (Fig. 5C), suggesting that the low-risk group might respond more effectively to immunotherapy.

### Prediction of immunotherapy benefits in BRCA patients

**Fig. 6:**
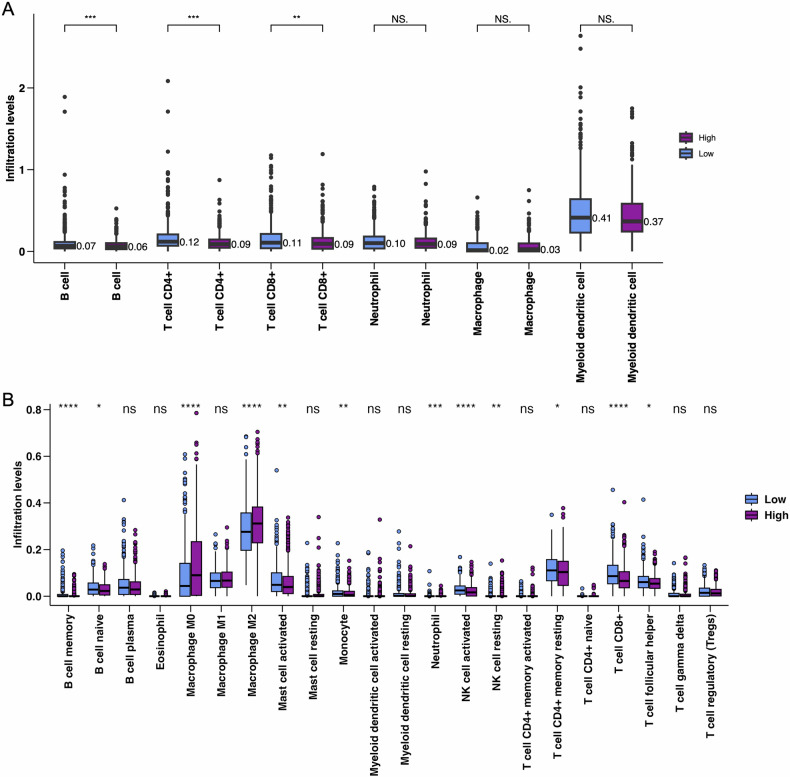
Immune cell infiltration analysis between high-BCIS and low-BCIS groups. **A** Comparison of total immune cell infiltration levels in different immune cell types between high-risk and low-risk groups. **B** Detailed infiltration levels of specific immune cell subsets. Statistical significance: *P < 0.05, **P < 0.01, ***P < 0.001, ns: not significant.

Additionally, this study conducted immune cell infiltration analysis using CIBERSORT and TIMER2.0. Overall, the high-risk group generally exhibited reduced immune cell infiltration. Notably, the infiltration of T cell CD8 + , B cell memory, and myeloid dendritic cell activated, which have direct tumor-killing effects, was significantly reduced in the high-risk group (Fig. 6A, B). Meanwhile, the pro-tumor macrophage M2 was significantly increased in the high-risk group (*p* < 0.0001). These results suggest that the immune environment in the high-risk group is more inclined to suppress anti-tumor immune responses, promote tumor growth, and facilitate immune evasion. This may lead to poorer prognosis and lower response to immunotherapy in high-risk group patients.

### Drug sensitivity analysis

**Fig. 7:**
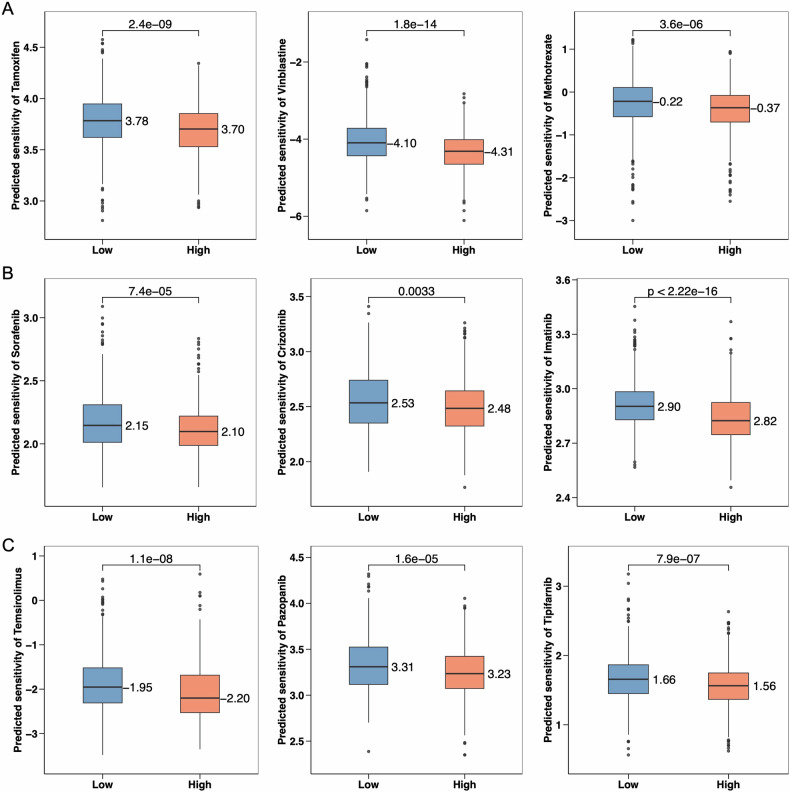
Predicted drug sensitivity analysis between high-BCIS and low-BCIS. **A** Sensitivity comparison of Tamoxifen, Vinblastine, and Methotrexate between high-BCIS and low-BCIS. **B** Sensitivity differences to Sorafenib, Crizotinib, and Imatinib between high-BCIS and low-BCIS. **C** Predicted sensitivity to Temsirolimus, Pazopanib, and Tipifarnib across high-BCIS and low-BCIS.

By conducting predictive analyses of drug sensitivity in different risk groups, we can better understand how patients respond to various medications. The study results indicate that the low-risk group has significantly higher sensitivity to the following drugs compared to the high-risk group: Tamoxifen (*p* = 2.4e-09), Vinblastine (*p* = 1.8e-14), Methotrexate (*p* = 3.6e-06), Sorafenib (*p* = 7.4e-05), Imatinib (*p* < 2.22e-16), Temsirolimus (*p* = 1.1e-08), Pazopanib (*p* = 1.6e-05), and Tipifarnib (Fig. 7A–C, *p* = 7.9e-07). Although there is a difference in sensitivity to Crizotinib between the high-risk and low-risk groups (*p* = 0.0033), this difference is relatively small, suggesting that both groups respond similarly to this drug.

On the other hand, high-risk patients show greater sensitivity to Cetuximab (*p* = 4.2e-06), Dabrafenib (*p* = 3e-09), and Erlotinib (*p* < 2.22e-16) (Fig. S5A–S5C). Dabrafenib is a BRAF inhibitor commonly used to treat BRAF V600E mutant melanoma, while Erlotinib is an EGFR inhibitor primarily used to treat non-small cell lung cancer with EGFR mutations. The high sensitivity to EGFR and BRAF pathways in the high-risk group may reflect these patients’ tumors’ heavy reliance on these signals. This suggests that using EGFR and BRAF inhibitors in high-risk patients may be more effective and help develop more personalized treatment strategies.

### PGK1 and PCMT1 in regulating PI3K/AKT/mTORC pathway activity

**Fig. 8:**
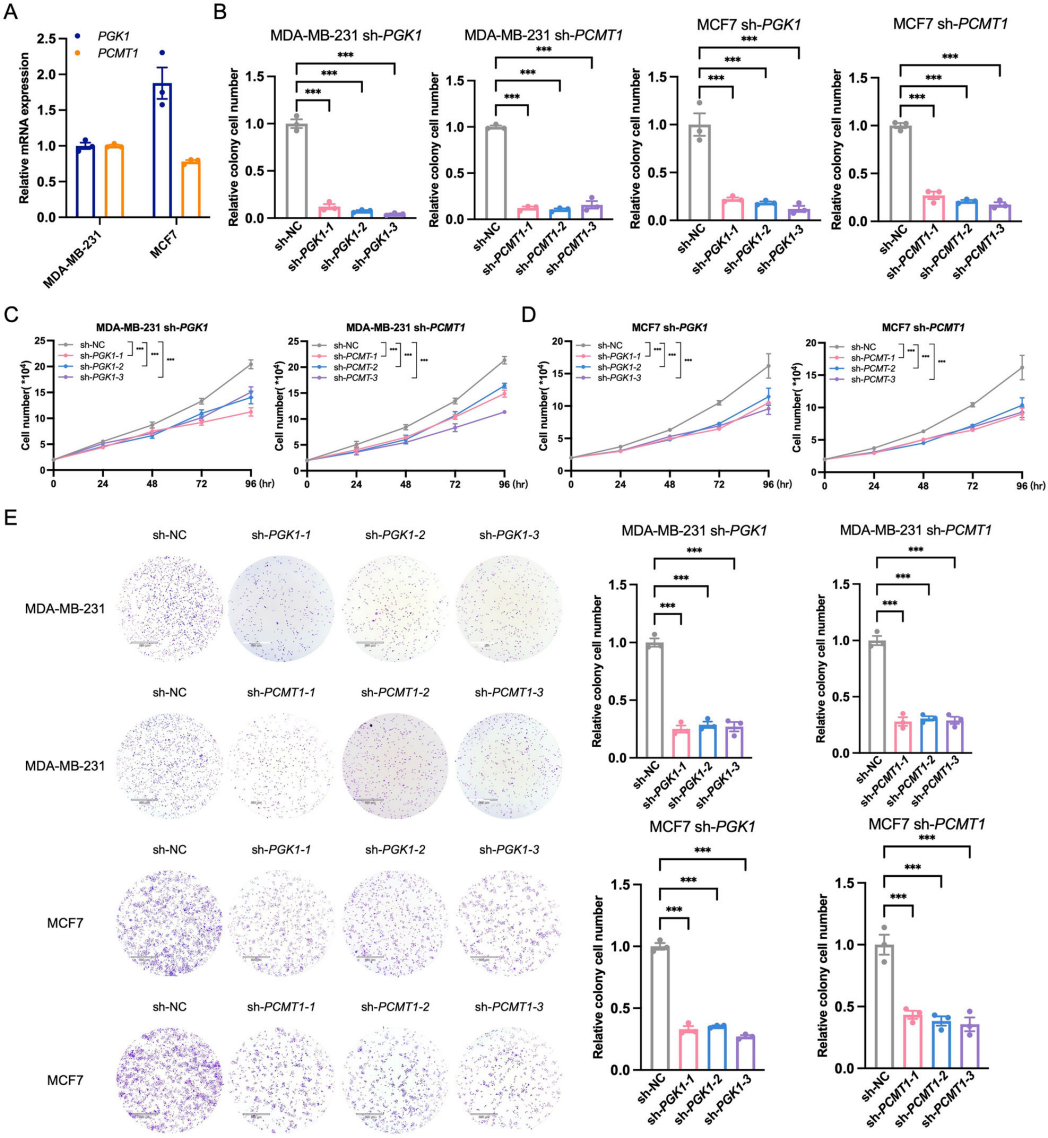
Impact of *PGK1* and *PCMT1* knockdown on PI3K/AKT/mTORC signaling in BC cell lines. **A** mRNA expression of *PGK1* and *PCMT1* in MCF7 and MDA-MB-231 cells. **B** Knockdown efficiency of *PGK1* and *PCMT1* genes in MCF7 and MDA-MB-231 using shRNAs. **C** Proliferation curves of MDA-MB-231 cells after *PGK1* and *PCMT1* gene knockdown. **D** Proliferation curves of MCF7 cells after *PGK1* and *PCMT1* gene knockdown. **E** Colony formation in MCF7 and MDA-MB-231 cells with *PGK1* and *PCMT1* knockdown. Statistical significance: *P < 0.05, **P < 0.01, ***P < 0.001.

This study aims to elucidate the role of *PGK1* and *PCMT1* in regulating the PI3K/AKT/mTORC signaling pathway in BC cells. First, the expression levels of *PGK1* and *PCMT1* in various BC cell lines were analyzed (Fig. 8A). Additionally, shRNA-mediated knockdown was performed in MCF7 and MDA-MB-231 cell lines to assess the impact of these genes on key cellular processes and pathway activities (Fig. 8B). Three shRNAs were designed to target *PGK1* and *PCMT1* in MCF7 and MDA-MB-231 cell lines, and the results showed that sh-*PGK1-1*, sh-*PGK1-2*, and sh-*PGK1-3* significantly suppressed PGK1 expression, while sh-*PCMT1-1*, sh-*PCMT1-2*, and sh-*PCMT1-3* significantly suppressed *PCMT1* expression. The shRNA-mediated knockdown experiments effectively inhibited the expression of *PGK1* and *PCMT1*, providing a basis for further understanding the specific roles of these two genes in BC cells. This process not only validated the effectiveness of gene knockdown but also provided evidence for evaluating the potential impact of *PGK1* and *PCMT1* on tumor cell growth and survival, thereby advancing research on their potential as therapeutic targets in BC.

To further investigate the biological roles of *PGK1* and *PCMT1* in BC, cell proliferation assays and colony formation analyses were conducted to evaluate the effects of *PGK1* and *PCMT1* knockdown on BC cell proliferation. The results of the cell proliferation assays indicated that the knockdown of *PGK1* and *PCMT1* significantly inhibited cell proliferation in the MCF7 and MDA-MB-231 cell lines. Compared to the control group, the cell proliferation rate was significantly reduced on fourth day following the knockdown of *PGK1* and *PCMT1*, suggesting that *PGK1* and *PCMT1* play a promotive role in the proliferation of BC cell lines (Fig. 8C, D). The colony formation assay also demonstrated a diminished cloning efficiency in cells with *PGK1* (*P* < 0.0001) and *PCMT1* (*P* < 0.0001) knockdown, underscoring the importance of these genes in supporting the survival and proliferative capacity of BC cells (Fig. 8E). The findings provide critical scientific evidence for the potential feasibility of targeting *PGK1* and *PCMT1* as therapeutic strategies in BC, aiding in the development of novel treatments that could enhance the precision and effectiveness of BC therapy.

**Fig. 9:**
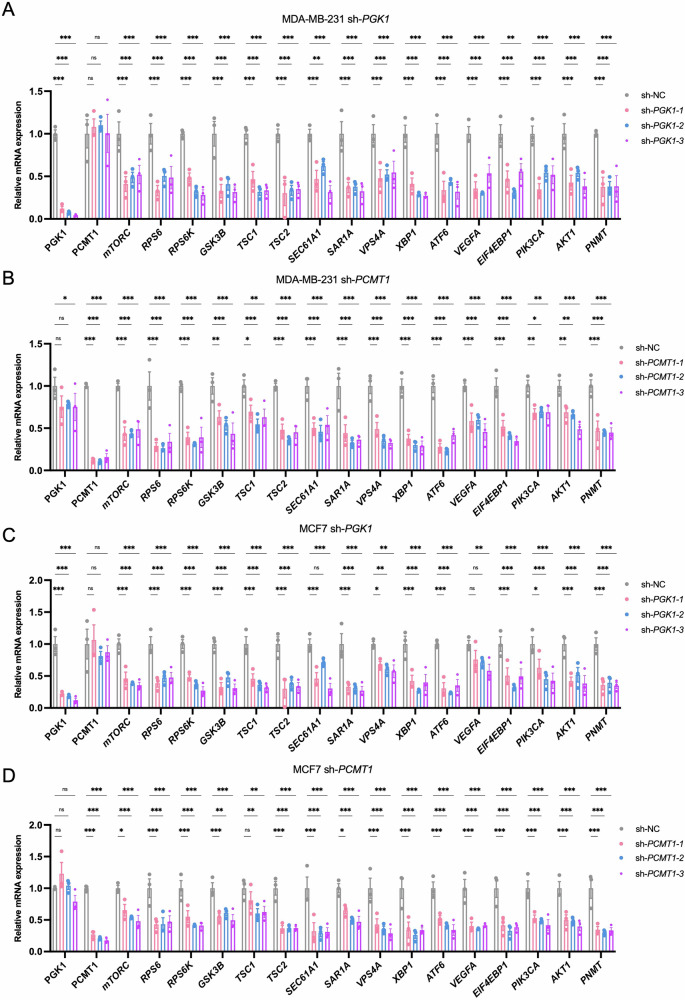
*PGK1* and *PCMT1* knockdown modulates PI3K/AKT/mTORC pathway activity in BC cell lines (MDA-MB-231 and MCF7). Relative mRNA expression levels of key genes involved in the PI3K/AKT/mTORC signaling pathway with *PGK1* (**A**) and *PCMT1* (**B**) knockdown in MDA-MB-231 cells. Relative mRNA expression levels of the same set of genes with *PGK1* (**C**) and *PCMT1* (**D**) knockdown in MCF7 cells. Statistical significance: *P < 0.05, **P < 0.01, ***P < 0.001, ns: not significant.

**Fig. 10:**
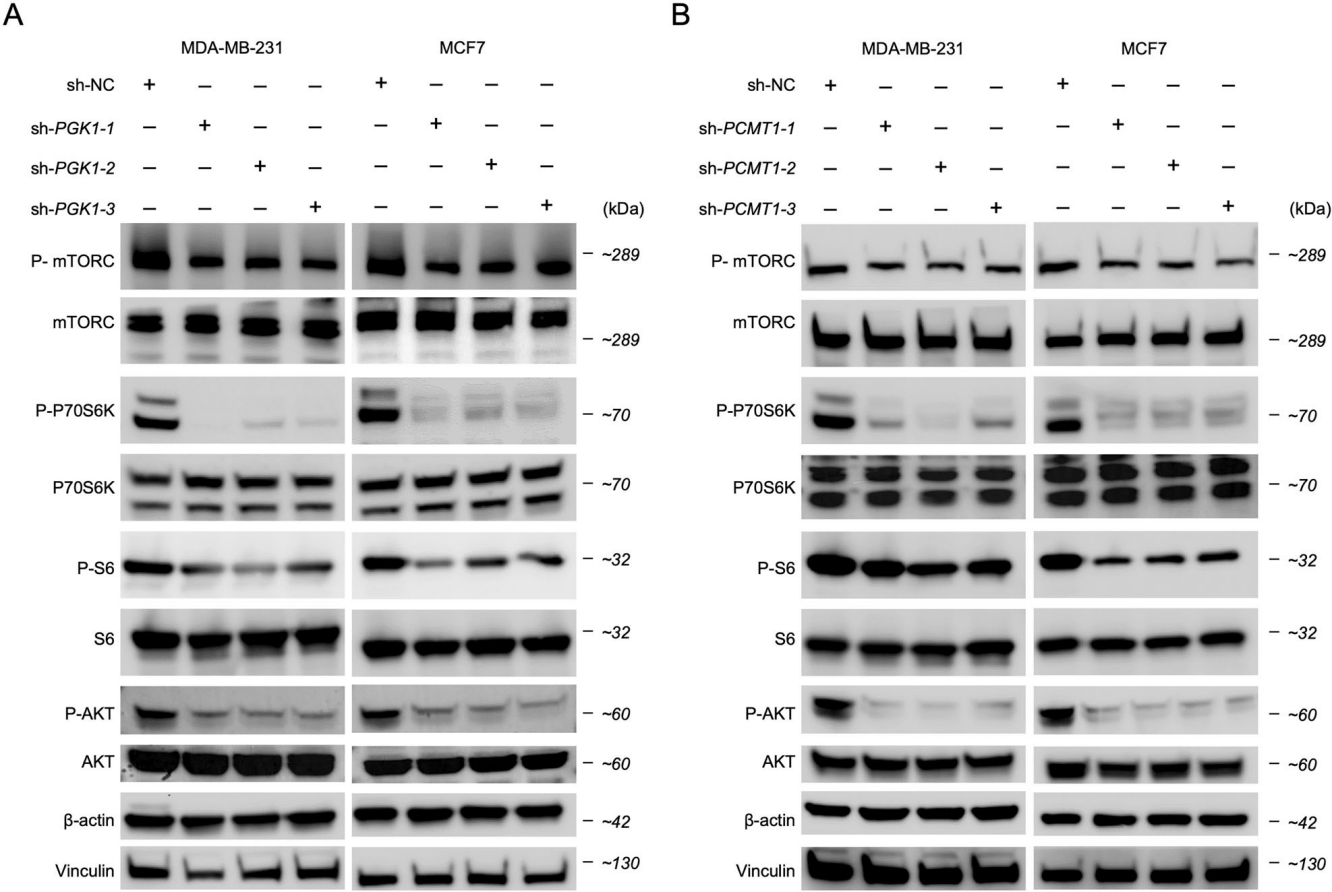
Western blot analysis of PI3K/AKT/mTORC signaling pathway marker genes in *PGK1* and *PCMT1* knockdown BC cell lines. **A** Western blot analysis of protein expression after *PGK1* knockdown in MDA-MB-231 and MCF7 cells. **B** Western blot analysis of protein expression after *PCMT1* knockdown in MDA-MB-231 and MCF7 cells.

Additionally, we focused on the relative expression levels of key genes involved in the PI3K/AKT/mTORC signaling pathway following the knockdown of *PGK1* and *PCMT1*. The results showed that shRNA-mediated knockdown of *PGK1* and *PCMT1* led to a significant reduction in the expression of multiple genes, including *mTORC*, *RPS6*, *RPS6K*, *GSK3B*, *TSC1*, *TSC2*, *SEC61A1*, *SAR1A*, *VPS4A*, *XBP1*, *ATF6*, *VEGFA*, *EIF4EBP1*, *PIK3CA*, *AKT1*, and *PNMT* in the MCF7 and MDA-MB-231 cell lines (Fig. 9A–D). Notably, the knockdown of *PGK1* and *PCMT1* had a consistent and significant impact on the expression of these genes, indicating that *PGK1* and *PCMT1* play a crucial role in maintaining the activity of PI3K/AKT/mTORC signaling pathway, which are essential for cell growth, survival, and proliferation. Finally, Western blot analysis using three different shRNAs to knock down *PGK1* and *PCMT1* led to reduction in the phosphorylation levels of key signaling proteins in both cell lines, including p-mTORC, p-P70S6K, p-S6 and p-AKT (Fig. 10A, B). Specifically, in the MDA-MB-231 BC cell line, the inhibitory effect on p-S6 protein was weaker when *PCMT1* was knocked down. Similarly, the expression level of p-mTORC did not show significant changes after *PCMT1* knockdown. This suggests that *PCMT1* may have a weaker role in the PI3K/AKT/mTORC signaling pathway or may regulate it indirectly through other pathways rather than directly.

This study reveals the critical role of *PGK1* and *PCMT1* in maintaining the PI3K/AKT/mTORC signaling pathway. It demonstrates the supportive role of *PGK1* and *PCMT1* in cell proliferation and survival, indicating their potential as therapeutic targets for BC. This finding contributes to a better understanding of the molecular mechanisms of BC and provides new directions for the development of precision therapeutic strategies.

## Discussion

This study analyzed intratumoral heterogeneity and molecular characteristics at single-cell resolution during the progression from DCIS to IDC_LM. By establishing risk score models with multiple markers and conducting larger-scale studies, we can better understand the progression mechanisms of DCIS and develop more effective clinical prediction tools.

Cellular heterogeneity is higher in the IDC and IDC_LM stages, reflecting the increasingly complex tumor microenvironment and the corresponding increase in cellular heterogeneity as the tumor progresses, especially during lymphatic metastasis.

However, we can still observe that the expression of *KRT8* in epithelial cells remains relatively stable during the transition from DCIS to IDC_LM. *KRT8* is mainly expressed in epithelial cells, indicating that epithelial cells play a key role in the development and progression of ductal carcinoma [24]. On the other hand, *CD2* is more likely to play a key role in the progression of ductal carcinoma, particularly in the metastasis of cancer from IDC to lymph nodes. Previous studies have shown that *CD2* on T cells is associated with directed migration, interacting with other molecules to help T cells respond to chemokine signals, thereby migrating to specific tissues or sites of inflammation [25]. *CD163* is often considered a marker of M2 macrophages, usually associated with anti-inflammatory and tissue repair responses [25]. It is expressed in samples from the DCIS and IDC stages but is not prominently present in the IDC_LM stage. In contrast, *MS4A1*, a marker for B cells, indicates a significant presence and possible functional activity of B cells in the IDC_LM. It is hypothesized that M2 macrophages may play an important role in the tumor microenvironment during the DCIS and IDC stages, while B cells may begin to play a more crucial role in tumor progression during the IDC_LM stage. This shift may reflect the different microenvironmental needs and immune evasion strategies of the tumor at different stages.

Epithelial cells are the primary cells of origin for BC [26]. During the DCIS stage, the malignancy remains confined to the epithelial cells within the breast ducts, although these cells already exhibit malignant characteristics. As the disease progresses, some epithelial cells undergo epithelial-mesenchymal transition (EMT), gaining invasiveness, breaching the basement membrane, and transitioning into IDC [27]. This process marks the progression of cancer from a localized lesion to an invasive disease [28]. As DCIS progresses to IDC, the gene expression profile of epithelial cells undergoes significant changes. These changes not only reflect the functional state of the cells but may also serve as biomarkers or therapeutic targets for disease progression. We regrouped epithelial cells in the samples and categorized them into nine subtypes based on the heterogeneity of gene expression within the cells. Interestingly, the composition of these subtypes changes significantly across different stages of DCIS and IDC. The differential genes of these subtypes could potentially serve as stage-specific biomarkers, allowing for more accurate identification of cancer progression stages and providing new therapeutic targets for developing stage-specific cancer therapies.

In the DCIS stage, the LumC1, LumC3, LumC4, and LumC6 subtypes occupy a significant proportion. Within the tumor microenvironment, *LGALS9* can both promote tumor growth through immune suppression and, under certain conditions, inhibit tumor growth [29]. LGALS9/Tim-3 is emerging as a novel cancer immunotherapy target. Additionally, recent studies have shown that *KRT15* is closely associated with tumorigenesis, with overexpression observed in squamous cell carcinoma samples [30] and a strong correlation with poor prognosis in colorectal cancer [31]. In another study, low *KRT15* expression was significantly associated with poor prognosis in BRCA patients [32]. *ALOX15B* has previously shown predictive value as a disulfide protease apoptosis-related prognostic feature in BC [33]. *CXCL13*, as a B-cell chemokine, has been shown to influence cancer cell proliferation, migration, and invasiveness in the tumor microenvironment [34, 35].

LumC2 is more closely associated with the invasiveness and metastasis of BC. *PAK1* has been shown to be hyperactive in various cancers and is directly linked to tumor invasion and metastasis [36–38]. Additionally, *BMPR1B* plays a critical role in BC progression by regulating the function of underlying proteins, serving as a diagnostic biomarker, and modulating the TGF-β and BMP signaling pathways [39]. LumC7 is dominant in the IDC stage but significantly reduced in the IDC_LM stage. Its differential genes, *DCD*, *IL32*, and *PPP1R1A*, may support tumor invasion and metastasis during the IDC stage. *IL32* expression has been associated with cancer pathways, cytokine-receptor interactions, and NOD-like receptor signaling pathways [40]. In previous studies, *IL32* has been identified as a potential biomarker for immune infiltration and poor prognosis, offering new therapeutic targets for cancer treatment [40]. However, during metastasis, changes in the tumor microenvironment and selective pressures lead to the reduced expression of these genes and the replacement of cell subtypes.

CNV scoring is used to assess genomic instability in tumors, which is generally associated with higher tumor invasiveness and poorer prognosis. In this study, we classified epithelial cells into invasion and non-invasion groups based on CNV scores and found that the proportion of the invasion group increases as cancer progresses. Differentially expressed genes include *MRPL43*, *EMC6*, *ASNA1*, *TCEA3*, *GSDMD*, *NAXE*, *SMIM7*, *NDUFA8*, *SSNA1*, and *HINT2*. Previous studies have shown that the upregulation of *MRPL43* increases the proliferation, invasion, and migration of colorectal cancer cell lines while reducing apoptosis [41]. Endoplasmic reticulum membrane protein complex subunit 6 (*EMC6*) plays an important role in both physiological and pathological states of cells [42, 43]. The upregulation of *EMC6* is associated with the proliferation, invasion, and migration of lung adenocarcinoma [43]. *NDUFA8* is highly expressed in cervical cancer tissues, and these levels are associated with reduced survival rates [44].

In this study, the BCIS prognostic model demonstrated high predictive power for patient prognosis in both the training and validation cohorts. To further explore the BCIS groups, GO pathway and GSEA assessments were conducted. We found that the high-BCIS group exhibited higher activity in the protein secretion signaling pathway, the PI3K/AKT/mTOR signaling pathway, and the *MTORC1* signaling pathway. Pathways or gene sets with high GSVA activity may serve as potential biomarkers for predicting disease progression, patient prognosis, or response to specific treatments.

Tumor evasion mechanisms that block the host’s immune response to tumor tissues are one of the characteristics identified in the Myeloid-Derived Suppressor Cell (MDSC) subpopulation, which induces tumor angiogenesis and immune evasion through T-cell suppression [45]. In this study, a higher MDSC score reflected a stronger immunosuppressive state. Additionally, patients with higher TIDE scores have a higher likelihood of immune evasion against antitumor immunity, and thus a lower response rate to ICB therapy [46]. The TIDE score has been shown to be more accurate than PD-L1 expression levels and TMB in predicting survival outcomes for cancer patients receiving ICB drugs [46–49]. Further research into the T-cell receptor (TCR) repertoire could provide additional insights into tumor immunity and potentially offer new biomarkers for predicting the efficacy of immunotherapy [50]. Overall, these observations suggest that patients classified in the high-NKGS group may face more aggressive tumor behavior and, due to the deeply immunosuppressive microenvironment, may encounter challenges in benefiting from immunotherapy.

The High-BCIS group exhibits higher levels of infiltration in some inhibitory or immune-evading cell types, which may be associated with a poorer prognosis. Correlations between the levels of immune cell infiltration of tumors and clinical outcomes have been investigated in many cancers [51]. The pattern of immune cell infiltration has become the only significant criterion, besides TNM staging, for predicting disease-free survival (DFS) and overall survival (OS) [52, 53].

The significance of studying drug sensitivity lies in understanding the differences in responses to specific drugs among patients in different risk groups, thereby providing a basis for personalized treatment. The half-maximal inhibitory concentration (IC50) is a commonly used indicator of drug sensitivity. It represents the concentration of a drug required to inhibit 50% of cell proliferation or biological activity in vitro [54]. A lower IC50 value indicates that the drug has a stronger inhibitory effect on cells, which is an ideal drug characteristic. Overall, the High-BCIS group shows significantly enhanced sensitivity to Tamoxifen and Vinblastine, while Methotrexate and Sorafenib may require more precise dose adjustments to achieve optimal therapeutic effects. By measuring the IC50 value of drugs, we can determine which patients are more sensitive to a particular drug, thereby optimizing treatment plans, reducing unnecessary side effects, and improving therapeutic outcomes.

Finally, BC, as a complex and heterogeneous disease, continues to be a significant health burden, with the PI3K/AKT/mTORC signaling pathway frequently implicated in its progression [55, 56]. This pathway is central to the regulation of cellular processes such as growth, proliferation, and survival, and its dysregulation is often observed in BC, contributing to aggressive disease characteristics and poor patient outcomes [57]. In the context of BC, *PGK1* and *PCMT1* have emerged as genes of interest. *PGK1*, a pivotal enzyme in glycolysis, has been associated with cancer metabolism, where it may support the high energy demands of rapidly dividing cancer cells [58]. Its elevated expression levels in various cancers, including BC, suggest a role in promoting tumor growth and metastasis. Previous studies and databases have reported that *PCMT1* expression is positively correlated with poor prognosis in several human cancers, including BC, bladder cancer, and endometrial cancer [59, 60]. *PCMT1* can interact and negatively regulate the tumor suppressor protein p53 (reduced protein level and activity) by carboxyl methylation of p53 at isoaspartate residues 29 and 30, which in turn represses apoptosis and growth arrest and contributes to cancer progression [61].

our study provides evidence that *PGK1* and *PCMT1* play a role in modulating the PI3K/AKT/mTORC signaling pathway in BC cells. The findings suggest that these genes may be integral to the malignant phenotype of BC and could be explored as targets for therapeutic intervention. Future research should focus on elucidating the molecular mechanisms by which *PGK1* and *PCMT1* interact with the PI3K/AKT/mTORC pathway and on evaluating the efficacy of targeted therapies in preclinical models of BC.

Overall, this study describes and validates 9 genes signature rooted in invasion-related cell genes, laying the foundation for personalized treatment strategies for BRCA patients. It was also found that high expression of *PGK1* and *PCMT1* is associated with upregulation of the PI3K/AKT/mTORC signaling pathway, leading to more aggressive tumor progression and, consequently, poorer prognosis. This study significantly advances the precision and applicability of biomarker discovery in BC. However, some limitations remain. The heterogeneity of BC patient samples means that individual differences may limit the generalizability of the risk score model, potentially affecting its predictive power in different populations or under varying experimental conditions. Additionally, although the function of biomarker genes can be confirmed during the experimental validation phase, in vitro results do not always fully reflect the complex biological environment in vivo. Moreover, the limited number of cell lines used in experimental validation may not comprehensively capture the diversity of BC. While the INAVO120 and CAPItello-291 studies focus on ER + , HER2- advanced breast cancer patients, our findings, although potentially valuable in prognosis prediction and experimentally validated, still require future clinical trials for validation [62–64].

## Methods and materials

### Cell culture

MCF7 or MDA-MB-231 cells were purchased from the Cell Bank of Chinese Academy of Sciences, Shanghai, China. Cells were cultured in Dulbecco’s Modified Eagle Medium (Gibco) supplemented with 10% FBS (Gibco) and 1% penicillin-streptomycin (Gibco) at 37 °C in a 5% CO_2_ humidified atmosphere.

### Cell proliferation assay

Cell proliferation was assessed using the Cell Counting Kit-8 (CCK8) assay. Transfected cells (sh-K, sh-T, and sh-NC) were seeded in 96-well plates at a density of 2 × 10^4^ cells per well and incubated for 24 h to allow attachment.

### Colony formation assay

Transfected MCF7 and MDA-MB-231 cells were seeded at 1500 or 500 cells per well in 6-well plates. The medium was changed every 3–4 days. After 14 days, colonies were fixed with 4% paraformaldehyde for 15 min and stained with 0.1% crystal violet for 30 min. Colonies consisting of at least 50 cells were counted manually. Each experiment was performed in triplicate. The cells were tested every two weeks for mycoplasma contamination. pLKO-shRNA was a gift from D. Anastasiou (Addgene, plasmid 42516), and scramble shRNA followed the sequences used in the experiments (Mission RNAi, Sigma), The primer sequences of mouse were as following in supplemental material.

### Western blotting

Cell were homogenized in lysis buffer (25 mM Tris-HCl pH 8.0, 150 mM NaCl, 1 mM CaCl2, 1% Triton X-100) with protease inhibitors (1:100, Bimake, B14001). The proteins concentration was determined. The proteins was separated using SDS-PAGE and then transferred to PVDF membranes (Millipore). The protein bands was blocked with 5% milk, incubated with antibodies and visualized by ECL (Proteintech). The following antibodies were used for western blotting: P-mTORC (CST, 5536 T), mTORC (CST, 2983 T), P-P70S6K (CST, 9234S), P70S6K (PTG, 66638-1-Ig), P-S6 (CST, 4858 s), S6 (CST, 2317 s), P-AKT (PTG,10176-2-AP), AKT (PTG,80455-1-RR), β-actin (PTG, HRP-66009), Vinculin (PTG, 66305-1-Ig).

### RNA isolation and quantitative PCR

Total RNA of 10 mg tissue was extracted using TRIzol (Invitrogen). RNA was purified by ethanol precipitation and reverse transcribed into cDNA using PrimeScriptTM RT Reagent Kit (Takara, RR047A). qPCR was performed on Fluorescence quantitative PCR instrument (ABI-7900-384) using TB Green Premix Ex TaqTMII (Takara, RR820A). Results are expressed as ΔΔCt values normalized to β-actin and graphed as relative transcript levels compared with controls. The primer sequences of mouse were as following in supplemental material. Primer sequences were list in Supplementary Table 1.

### Collection of single-cell RNA sequencing and GEO datasets

This study performed a comprehensive analysis of the scRNA-seq dataset GSE195861 and constructed a cellular atlas based on it [14]. The dataset contains 20 tissue samples, including 7 from patients diagnosed with DCIS, 6 from IDC patients, and 6 from IDC_LM samples. Additionally, we obtained one normal breast tissue sample from a DCIS patient who underwent a mastectomy to exclude the mixing of normal cells in DCIS and IDC samples. The raw read counts and associated clinical details such as age, gender, stage, overall survival (OS), and vital status for BRCA patients were accessed from the UCSC Xena website (https://xenabrowser.net/datapages/). To further validate the OS status of the proposed gene set, we refer to data and clinical details of GSE20685 from the NCBI GEO database (*n* = 327), as well as five years and ten years survival data from the METABRIC dataset.

### Single cell analysis and cell clustering

An extensive analysis of the single-cell dataset matrix was conducted using Scanpy (version 1.9.1). First, dimensionality reduction was performed through Principal Component Analysis (PCA), with “ov.pp.pca” set to “n_pcs=50” to capture the primary variations in the data. Next, based on the standardized and PCA-processed data, the neighborhood relationships between cells were calculated, with “n_neighbors=15” to construct a neighborhood graph, laying the foundation for subsequent clustering analysis. Then, the Leiden clustering method was applied to identify cell clusters, with “resolution=0.2” to ensure an appropriate resolution of the clusters. Finally, the clustering results were visualized using “sc.pl.umap” presenting the cell clusters in UMAP form. Additionally, the CellTypist tool was used to annotate cell types based on known marker genes, providing a basis for further biological interpretation.

### Construction and validation of the prognostic signature

Using univariate Cox regression analysis, we screened for marker genes that are significantly associated with overall survival (OS) in TCGA-BRCA patients between the invasion and non-invasion cell groups, establishing an initial prognostic model. We set *P* < 0.05 as the threshold for selecting prognostic genes. Next, we employed the least absolute shrinkage and selection operator (LASSO) Cox proportional hazards regression model using the “glmnet” R package to further refine these initially screened genes and identify those with the greatest impact on prognosis [65]. Subsequently, we constructed a final risk score model by linearly combining the mRNA expression levels of the selected genes with their corresponding risk coefficients. Through this model, we identified nine key candidate prognosis-related genes and classified patients into high-BCIS and low-BCIS groups based on the median risk score. Finally, we evaluated the predictive capability and clinical utility of the BCIS model by constructing receiver operating characteristic (ROC) curves.

### Survival analysis

The high-risk group is significantly associated with poorer overall survival (OS). To validate this, we utilized the ‘survival’ and ‘survminer’ R packages to analyze the expression of genes related to the BCIS and their prognostic relevance in the TCGA-BRCA dataset using Kaplan-Meier (KM) curves. Furthermore, we conducted survival analysis on the five years and ten years survival data from METABRIC, as well as the GSE20685 dataset, to confirm the predictive capability of the BCIS model.

### Differential expressed gene analysis

The expression levels of individual genes within each cluster were compared to the remaining cells using the “sc.tl.rank_genes_groups” module and the Wilcoxon rank-sum test. A gene was defined as upregulated or downregulated based on a significance threshold of *P* < 0.05, with cutoff criteria of log (fold change) ≥ 2 or ≤ -2, respectively.

### Gene set enrichment analysis (GSEA)

Gene Set Enrichment Analysis (GSEA) was conducted using the hallmark gene sets (h.all.v2023.2.Hs.symbols.gmt) from MSigDB (http://software.broadinstitute.org/gsea/msigdb/) to identify significantly enriched pathways.

### Gene set variation analysis (GSVA)

Gene Set Variation Analysis (GSVA) was carried out to discern the activity of enriched pathways between the high-BCIS and low-BCIS groups. The enrichment scores for each gene set in the TCGA-BRCA samples were determined using the ssGSEA algorithm via the “fgsea” R package.

### Multi-dimensional evaluation of comprehensive tumor microenvironment and drug sensitivity

In this study, we utilized the ESTIMATE and Tumor Immune Dysfunction and Exclusion (TIDE) algorithm in R to assess the infiltration levels of immune and stromal cells in the tumor microenvironment, the purity of tumor samples, and the potential for immune evasion and treatment response prediction [46]. TCR analysis, as a key method, was employed to study and evaluate the diversity, specificity, and immune response of T cells, with a detailed analysis of TCR diversity and richness based on previous research [66]. Additionally, TIMER2.0 and CIBERSORT were used to estimate the proportions of different immune cell types within tumor samples, thereby revealing the infiltration levels of these cells. Finally, based on drug sensitivity prediction datasets ‘PANCANCER_IC_Tue_Aug_9_15_28_57_2016’ and ‘cgp2016ExprRma’, as well as the database ‘drugData2016’ used for extracting and filtering drug data related to specific tissue types, we employed the pRRophetic package to predict drug sensitivity. The integration of these tools provided significant support for our in-depth understanding of the tumor microenvironment and its potential response to treatment.

### Statistics analysis

Differences of statistical significance were evaluated using a two-tailed Student’s t-test on the R platform. Multivariate analysis employing the Cox proportional hazards model was executed using the R packages (“survival”, “survminer”, and “forestplot”) to pinpoint independent factors linked to OS in both TCGA-BRCA, GEO and METABRIC cohorts. The *P* value was corrected using the false discovery rate (FDR), with values or FDR < 0.05 deemed significant. An adjusted *P* < 0.05 served as the threshold criterion.

In addition, data are presented as mean ± SEM. Statistical significance was analysed using the unpaired two-tailed Student’s *t*-test at least 3 independent experiments using GraphPad Prism (GraphPad Software, USA). *P*-value < 0.05 was considered statistically significant. *P < 0.05; **P < 0.01; ***P < 0.001.

## Supplementary information

Supplementary figure and table legends

Figure S1

Figure S2

Figure S3

Figure S4

Figure S5

Supplementary Table 1

Western Blot

## Data Availability

The single-cell RNA-seq dataset was sourced from the GEO database using the accession number GSE195861. We obtained the TCGA-BRCA cohort dataset from The Cancer Genome Atlas (TCGA) portal (https://portal.gdc.cancer.gov/). Additionally, for validation purposes, we retrieved datasets GSE20685 from the GEO database, and obtained five years and ten years survival data for METABRIC.

## Code availability

The codes used in this paper are available from the lead contact upon request.

## References

[1] Siegel RL, Miller KD, Fuchs HE, Jemal A. Cancer statistics, 2022. CA Cancer J Clin. 2022;72:7–33. 35020204 10.3322/caac.21708

[2] Arnold M, Morgan E, Rumgay H, Mafra A, Singh D, Laversanne M, et al. Current and future burden of breast cancer: Global statistics for 2020 and 2040. Breast. 2022;66:15–23. 36084384 10.1016/j.breast.2022.08.010 PMC9465273

[3] Sharma R. Global, regional, national burden of breast cancer in 185 countries: evidence from GLOBOCAN 2018. Breast Cancer Res Treat. 2021;187:557–67. 33515396 10.1007/s10549-020-06083-6

[4] Sung H, Ferlay J, Siegel RL, Laversanne M, Soerjomataram I, Jemal A, et al. Global Cancer Statistics 2020: GLOBOCAN Estimates of Incidence and Mortality Worldwide for 36 Cancers in 185 Countries. CA Cancer J Clin. 2021;71:209–49. 33538338 10.3322/caac.21660

[5] Wilson GM, Dinh P, Pathmanathan N, Graham JD. Ductal Carcinoma in Situ: Molecular Changes Accompanying Disease Progression. J Mammary Gland Biol Neoplasia. 2022;27:101–31. 35567670 10.1007/s10911-022-09517-7 PMC9135892

[6] Knowlton CA, Jimenez RB, Moran MS. DCIS: Risk Assessment in the Molecular Era. Semin Radiat Oncol. 2022;32:189–97. 35688517 10.1016/j.semradonc.2022.01.005

[7] van Seijen M, Lips EH, Thompson AM, Nik-Zainal S, Futreal A, Hwang ES, et al. Ductal carcinoma in situ: to treat or not to treat, that is the question. Br J Cancer. 2019;121:285–92. 31285590 10.1038/s41416-019-0478-6 PMC6697179

[8] Goh CW, Wu J, Ding S, Lin C, Chen X, Huang O, et al. Invasive ductal carcinoma with coexisting ductal carcinoma in situ (IDC/DCIS) versus pure invasive ductal carcinoma (IDC): a comparison of clinicopathological characteristics, molecular subtypes, and clinical outcomes. J Cancer Res Clin Oncol. 2019;145:1877–86. 31089799 10.1007/s00432-019-02930-2 PMC11810233

[9] Hannafon BN, Ding WQ. miRNAs as Biomarkers for Predicting the Progression of Ductal Carcinoma in Situ. Am J Pathol. 2018;188:542–9. 29246496 10.1016/j.ajpath.2017.11.003 PMC5840484

[10] Schoppmann SF, Horvat R, Birner P. Lymphatic vessels and lymphangiogenesis in female cancer: mechanisms, clinical impact and possible implications for anti-lymphangiogenic therapies (Review). Oncol Rep. 2002;9:455–60. 11956609

[11] Duffy MJ, McDermott EW, Crown J. Use of Multiparameter Tests for Identifying Women with Early Breast Cancer Who Do Not Need Adjuvant Chemotherapy. Clin Chem. 2017;63:804–6. 28188230 10.1373/clinchem.2016.267161

[12] Duffy MJ, O’Donovan N, McDermott E, Crown J. Validated biomarkers: The key to precision treatment in patients with breast cancer. Breast. 2016;29:192–201. 27521224 10.1016/j.breast.2016.07.009

[13] Glencer AC, Miller PN, Greenwood H, Rodas CKM, Freimanis R, Basu A, et al. Identifying Good Candidates for Active Surveillance of Ductal Carcinoma In Situ: Insights from a Large Neoadjuvant Endocrine Therapy Cohort. Cancer Res Commun. 2022;2:1579–89. 36970720 10.1158/2767-9764.CRC-22-0263 PMC10035518

[14] Tokura M, Nakayama J, Prieto-Vila M, Shiino S, Yoshida M, Yamamoto T, et al. Single-Cell Transcriptome Profiling Reveals Intratumoral Heterogeneity and Molecular Features of Ductal Carcinoma In Situ. Cancer Res. 2022;82:3236–48. 35852797 10.1158/0008-5472.CAN-22-0090

[15] Moelans CB, Verschuur-Maes AH, van Diest PJ. Frequent promoter hypermethylation of BRCA2, CDH13, MSH6, PAX5, PAX6 and WT1 in ductal carcinoma in situ and invasive breast cancer. J Pathol. 2011;225:222–31. 21710692 10.1002/path.2930

[16] Lambein K, Van Bockstal M, Vandemaele L, Van den Broecke R, Cocquyt V, Geenen S, et al. Comparison of HER2 amplification status among breast cancer subgroups offers new insights in pathways of breast cancer progression. Virchows Arch. 2017;471:575–87. 28567637 10.1007/s00428-017-2161-8

[17] Tewari D, Patni P, Bishayee A, Sah AN, Bishayee A. Natural products targeting the PI3K-Akt-mTOR signaling pathway in cancer: A novel therapeutic strategy. Semin Cancer Biol. 2022;80:1–17. 31866476 10.1016/j.semcancer.2019.12.008

[18] Song Q, Zhang W, Shi D, Zhang Z, Zhao Q, Wang M, et al. Overexpression of cannabinoid receptor 2 is associated with human breast cancer proliferation, apoptosis, chemosensitivity and prognosis via the PI3K/Akt/mTOR signaling pathway. Cancer Med. 2023;12:13538–50. 37220224 10.1002/cam4.6037 PMC10315729

[19] Dey N, De P, Leyland-Jones B. PI3K-AKT-mTOR inhibitors in breast cancers: From tumor cell signaling to clinical trials. Pharmacol Ther. 2017;175:91–106. 28216025 10.1016/j.pharmthera.2017.02.037

[20] Ding S, Chen X, Shen K. Single-cell RNA sequencing in breast cancer: Understanding tumor heterogeneity and paving roads to individualized therapy. Cancer Commun (Lond). 2020;40:329–44. 32654419 10.1002/cac2.12078 PMC7427308

[21] Wolf FA, Angerer P, Theis FJ. SCANPY: large-scale single-cell gene expression data analysis. Genome Biol. 2018;19:15. 29409532 10.1186/s13059-017-1382-0 PMC5802054

[22] Zeng Z, Ma Y, Hu L, Tan B, Liu P, Wang Y, et al. OmicVerse: a framework for bridging and deepening insights across bulk and single-cell sequencing. Nat Commun. 2024;15:5983. 39013860 10.1038/s41467-024-50194-3 PMC11252408

[23] Cao Y, Wang X, Peng G. SCSA: A Cell Type Annotation Tool for Single-Cell RNA-seq Data. Front Genet. 2020;11:490. 32477414 10.3389/fgene.2020.00490 PMC7235421

[24] Xie L, Dang Y, Guo J, Sun X, Xie T, Zhang L, et al. High KRT8 Expression Independently Predicts Poor Prognosis for Lung Adenocarcinoma Patients. Genes (Basel). 2019;10:36. 30634629 10.3390/genes10010036 PMC6360019

[25] Romain G, Strati P, Rezvan A, Fathi M, Bandey IN, Adolacion JRT, et al. Multidimensional single-cell analysis identifies a role for CD2-CD58 interactions in clinical antitumor T cell responses. J Clin Invest. 2022;132:e159402. 35881486 10.1172/JCI159402 PMC9433104

[26] Polyak K. Heterogeneity in breast cancer. J Clin Invest. 2011;121:3786–8. 21965334 10.1172/JCI60534 PMC3195489

[27] Lakhtakia R, Aljarrah A, Furrukh M, Ganguly SS. Epithelial Mesenchymal Transition (EMT) in Metastatic Breast Cancer in Omani Women. Cancer Microenviron. 2017;10:25–37. 28526992 10.1007/s12307-017-0194-9 PMC5750198

[28] Etzerodt A, Moestrup SK. CD163 and inflammation: biological, diagnostic, and therapeutic aspects. Antioxid Redox Signal. 2013;18:2352–63. 22900885 10.1089/ars.2012.4834 PMC3638564

[29] Lv Y, Ma X, Ma Y, Du Y, Feng J. A new emerging target in cancer immunotherapy: Galectin-9 (LGALS9). Genes Dis. 2023;10:2366–82. 37554219 10.1016/j.gendis.2022.05.020 PMC10404877

[30] Sanchez-Palencia A, Gomez-Morales M, Gomez-Capilla JA, Pedraza V, Boyero L, Rosell R, et al. Gene expression profiling reveals novel biomarkers in nonsmall cell lung cancer. Int J Cancer. 2011;129:355–64. 20878980 10.1002/ijc.25704

[31] Rao X, Wang J, Song HM, Deng B, Li JG. KRT15 overexpression predicts poor prognosis in colorectal cancer. Neoplasma. 2020;67:410–4. 31884802 10.4149/neo_2019_190531N475

[32] Zhong P, Shu R, Wu H, Liu Z, Shen X, Hu Y. Low KRT15 expression is associated with poor prognosis in patients with breast invasive carcinoma. Exp Ther Med. 2021;21:305. 33717248 10.3892/etm.2021.9736 PMC7885068

[33] Wang Z, Du X, Lian W, Chen J, Hong C, Li L, et al. A novel disulfidptosis-associated expression pattern in breast cancer based on machine learning. Front Genet. 2023;14:1193944. 37456667 10.3389/fgene.2023.1193944 PMC10343428

[34] Kazanietz MG, Durando M, Cooke M. CXCL13 and Its Receptor CXCR5 in Cancer: Inflammation, Immune Response, and Beyond. Front Endocrinol (Lausanne). 2019;10:471. 31354634 10.3389/fendo.2019.00471 PMC6639976

[35] Wang B, Wang M, Ao D, Wei X. CXCL13-CXCR5 axis: Regulation in inflammatory diseases and cancer. Biochim Biophys Acta Rev Cancer. 2022;1877:188799. 36103908 10.1016/j.bbcan.2022.188799

[36] Wang S, Wang SY, Du F, Han Q, Wang EH, Luo EJ, et al. Knockdown of PAK1 Inhibits the Proliferation and Invasion of Non-Small Cell Lung Cancer Cells Through the ERK Pathway. Appl Immunohistochem Mol Morphol. 2020;28:602–10. 31394555 10.1097/PAI.0000000000000803

[37] Yi Q, Chen T, Zhou K, Ma Q, Sun Z, Shi H. PAK1 Inhibition Suppresses the Proliferation, Migration and Invasion of Glioma Cells. Curr Protein Pept Sci. 2023;24:178–89. 36573046 10.2174/1389203724666221226150329

[38] Cao F, Yin LX. PAK1 promotes proliferation, migration and invasion of hepatocellular carcinoma by facilitating EMT via directly up-regulating Snail. Genomics. 2020;112:694–702. 31071459 10.1016/j.ygeno.2019.05.002

[39] Azadeh M, Salehzadeh A, Ghaedi K, Sasani ST. Integrated High-Throughput Bioinformatics (Microarray, RNA-Seq, and RNA Interaction) and qRT-PCR Investigation of BMPR1B Axis as a Potential Diagnostic Biomarker of Isfahan Breast Cancer. Adv Biomed Res. 2023;12:120. 37434942 10.4103/abr.abr_200_22 PMC10331528

[40] Han F, Ma J. Pan-cancer analysis reveals IL32 is a potential prognostic and immunotherapeutic biomarker in cancer. Sci Rep. 2024;14:8129. 38584169 10.1038/s41598-024-58550-5 PMC10999427

[41] Zhu X, Liu Y, Xu J, Cheng Z, Yu Y, Chu M, et al. miR-608 rs4919510 Polymorphism May Affect Susceptibility to Colorectal Cancer by Upregulating MRPL43 Expression. DNA Cell Biol. 2020;39:2017–27. 33147064 10.1089/dna.2020.5689

[42] Tan JH, Cao RC, Zhou L, Zhou ZT, Chen HJ, Xu J, et al. EMC6 regulates acinar apoptosis via APAF1 in acute and chronic pancreatitis. Cell Death Dis. 2020;11:966. 33177505 10.1038/s41419-020-03177-3 PMC7658364

[43] Zhou X, Xiao B, Jiang M, Rui J. Pan-cancer analysis identifies EMC6 as a potential target for lung adenocarcinoma. iScience. 2024;27:108648. 38155776 10.1016/j.isci.2023.108648 PMC10753071

[44] Xiang H, Tang H, He Q, Sun J, Yang Y, Kong L, et al. NDUFA8 is transcriptionally regulated by EP300/H3K27ac and promotes mitochondrial respiration to support proliferation and inhibit apoptosis in cervical cancer. Biochem Biophys Res Commun. 2024;693:149374. 38096616 10.1016/j.bbrc.2023.149374

[45] Gallamini A, Raimondo FD, Nasa GL, Romano A, Borra A, Greco M. Standard therapies versus novel therapies in Hodgkin lymphoma. Immunol Lett. 2013;155:56–9. 24140162 10.1016/j.imlet.2013.09.011

[46] Jiang P, Gu S, Pan D, Fu J, Sahu A, Hu X, et al. Signatures of T cell dysfunction and exclusion predict cancer immunotherapy response. Nat Med. 2018;24:1550–8. 30127393 10.1038/s41591-018-0136-1 PMC6487502

[47] Wang S, He Z, Wang H, Li H, Liu XS. Antigen presentation and tumor immunogenicity in cancer immunotherapy response prediction. Elife. 2019;8:e49020. 10.7554/eLife.49020 PMC6879305 31767055

[48] Keenan TE, Burke kp, Van Allen EM. Genomic correlates of response to immune checkpoint blockade. Nat Med. 2019;25:389–402. 30842677 10.1038/s41591-019-0382-x PMC6599710

[49] Kaderbhai C, Tharin Z, Ghiringhelli F. The Role of Molecular Profiling to Predict the Response to Immune Checkpoint Inhibitors in Lung Cancer. Cancers (Basel). 2019;11:201. 30744168 10.3390/cancers11020201 PMC6406957

[50] Yang H, Wang Y, Jia Z, Wang Y, Yang X, Wu P, et al. Characteristics of T-Cell Receptor Repertoire and Correlation With EGFR Mutations in All Stages of Lung Cancer. Front Oncol. 2021;11:537735. 33777727 10.3389/fonc.2021.537735 PMC7991722

[51] Fridman WH, Pagès F, Sautès-Fridman C, Galon J. The immune contexture in human tumours: impact on clinical outcome. Nat Rev Cancer. 2012;12:298–306. 22419253 10.1038/nrc3245

[52] Mlecnik B, Tosolini M, Kirilovsky A, Berger A, Bindea G, Meatchi T, et al. Histopathologic-based prognostic factors of colorectal cancers are associated with the state of the local immune reaction. J Clin Oncol. 2011;29:610–8. 21245428 10.1200/JCO.2010.30.5425

[53] Broussard EK, Disis ML. TNM staging in colorectal cancer: T is for T cell and M is for memory. J Clin Oncol. 2011;29:601–3. 21245434 10.1200/JCO.2010.32.9078

[54] Park A, Joo M, Kim K, Son WJ, Lim G, Lee J, et al. A comprehensive evaluation of regression-based drug responsiveness prediction models, using cell viability inhibitory concentrations (IC50 values). Bioinformatics. 2022;38:2810–7. 35561188 10.1093/bioinformatics/btac177

[55] Glaviano A, Foo ASC, Lam HY, Yap KCH, Jacot W, Jones RH, et al. PI3K/AKT/mTOR signaling transduction pathway and targeted therapies in cancer. Mol Cancer. 2023;22:138. 37596643 10.1186/s12943-023-01827-6 PMC10436543

[56] Li H, Prever L, Hirsch E, Gulluni F. Targeting PI3K/AKT/mTOR Signaling Pathway in Breast Cancer. Cancers (Basel). 2021;13:3517. 34298731 10.3390/cancers13143517 PMC8304822

[57] Bahrami A, Khazaei M, Shahidsales S, Hassanian SM, Hasanzadeh M, Maftouh M, et al. The Therapeutic Potential of PI3K/Akt/mTOR Inhibitors in Breast Cancer: Rational and Progress. J Cell Biochem. 2018;119:213–22. 28513879 10.1002/jcb.26136

[58] Duncan L, Shay C, Teng Y. PGK1 : An Essential Player in Modulating Tumor Metabolism. Methods Mol Biol. 2022;2343:57–70. 34473315 10.1007/978-1-0716-1558-4_4

[59] Amer M, Elhefnawi M, El-Ahwany E, Awad AF, Gawad NA, Zada A, et al. Hsa-miR-195 targets PCMT1 in hepatocellular carcinoma that increases tumor life span. Tumour Biol. 2014;35:11301–9. 25119594 10.1007/s13277-014-2445-4

[60] Dong L, Li Y, Xue D, Liu T. PCMT1 is an unfavorable predictor and functions as an oncogene in bladder cancer. IUBMB Life. 2018;70:291–9. 29517839 10.1002/iub.1717

[61] Lee JC, Kang SU, Jeon Y, Park JW, You JS, Ha SW, et al. Protein L-isoaspartyl methyltransferase regulates p53 activity. Nat Commun. 2012;3:927. 22735455 10.1038/ncomms1933 PMC3621463

[62] Turner NC, Oliveira M, Howell SJ, Dalenc F, Cortes J, Moreno HLG, et al. Capivasertib in Hormone Receptor-Positive Advanced Breast Cancer. N Engl J Med. 2023;388:2058–70. 37256976 10.1056/NEJMoa2214131 PMC11335038

[63] Gradishar WJ. Improving the Outcome of Bad-Acting Hormone Receptor-Positive Breast Cancer. N Engl J Med. 2024;391:1644–7. 39476346 10.1056/NEJMe2411229

[64] Turner NC, Im SA, Saura C, Juric D, Loibl S, Kalinsky K, et al. Inavolisib-Based Therapy in PIK3CA-Mutated Advanced Breast Cancer. N Engl J Med. 2024;391:1584–96. 39476340 10.1056/NEJMoa2404625

[65] Engebretsen S, Bohlin J. Statistical predictions with glmnet. Clin Epigenetics. 2019;11:123. 31443682 10.1186/s13148-019-0730-1 PMC6708235

[66] Thorsson V, Gibbs DL, Brown SD, Wolf D, Bortone DS, Yang THO, et al. The Immune Landscape of Cancer. Immunity. 2018;48:812–30. 29628290 10.1016/j.immuni.2018.03.023 PMC5982584

