# Supplementary material for: Development of a breast cancer invasion score to predict tumor aggressiveness and prognosis via PI3K/AKT/mTOR pathway analysis
Source: Cell Death Discov. 2025 Apr 9;11:157. doi: 10.1038/s41420-025-02422-y (PMC11982538; doi:10.1038/s41420-025-02422-y)
Supplement: Supplementary file 1 — Supplementary figure and table legends [file 41420_2025_2422_MOESM1_ESM.docx]

**Supplementary figure and table legends**

**Supplementary Figure 1.** (A) UMAP plot of epithelial cells, colored by 20 samples. (B) The correlation between different cell types. Color indicates the strength of the correlation, from green (negative correlation) to orange (positive correlation). (C) Characteristic gene expression distribution of four cell types in UMAP plot. (D) Heatmap of differential gene expression in different cell types.

**Supplementary Figure 2.** (A) UMAP plot of epithelial cells, colored by 20 samples. (B) UMAP plot of epithelial cells, colored by Normal, DCIS, IDC and IDC_LM. (C) Expression levels of differentially expressed genes in different stages of ductal carcinoma. (D) Heatmap of differential gene expression in nine luminal subtypes.

**Supplementary Figure 3.** (A) Expression level of different genes in invasive and non-invasive group. (B) Heatmap of the expression distribution of differential genes in different stages of ductal carcinoma in invasive and non-invasive groups. (C) Heatmap of differential gene expression between invasive and non-invasive groups. (D) Violin plot of the expression distribution of the nine most predictive differential genes in the risk prediction model at different stages of ductal carcinoma. (E) Network plot of 8 invasive signature genes and related genes.

**Supplementary Figure 4.** Tumor microenvironment analysis between high-BCIS and low-BCIS groups. TIDE analysis in high-BCIS and low-BCIS groups, including (A) CAF and (B) M2.

**Supplementary Figure 5.** Predicted Drug Sensitivity Analysis Between high-BCIS and low-BCIS. (A) Sensitivity comparison of (A) Cetuximab, (B) Dabrafenib and (C) Erlotinib between high-BCIS and low-BCIS.

**Supplementary Table 1.** Primer sequences used for the validation of all selected genes.
