# Supplementary figures and images for: Development of a breast cancer invasion score to predict tumor aggressiveness and prognosis via PI3K/AKT/mTOR pathway analysis
Source: Cell Death Discov. 2025 Apr 9;11:157. doi: 10.1038/s41420-025-02422-y (PMC11982538; doi:10.1038/s41420-025-02422-y)

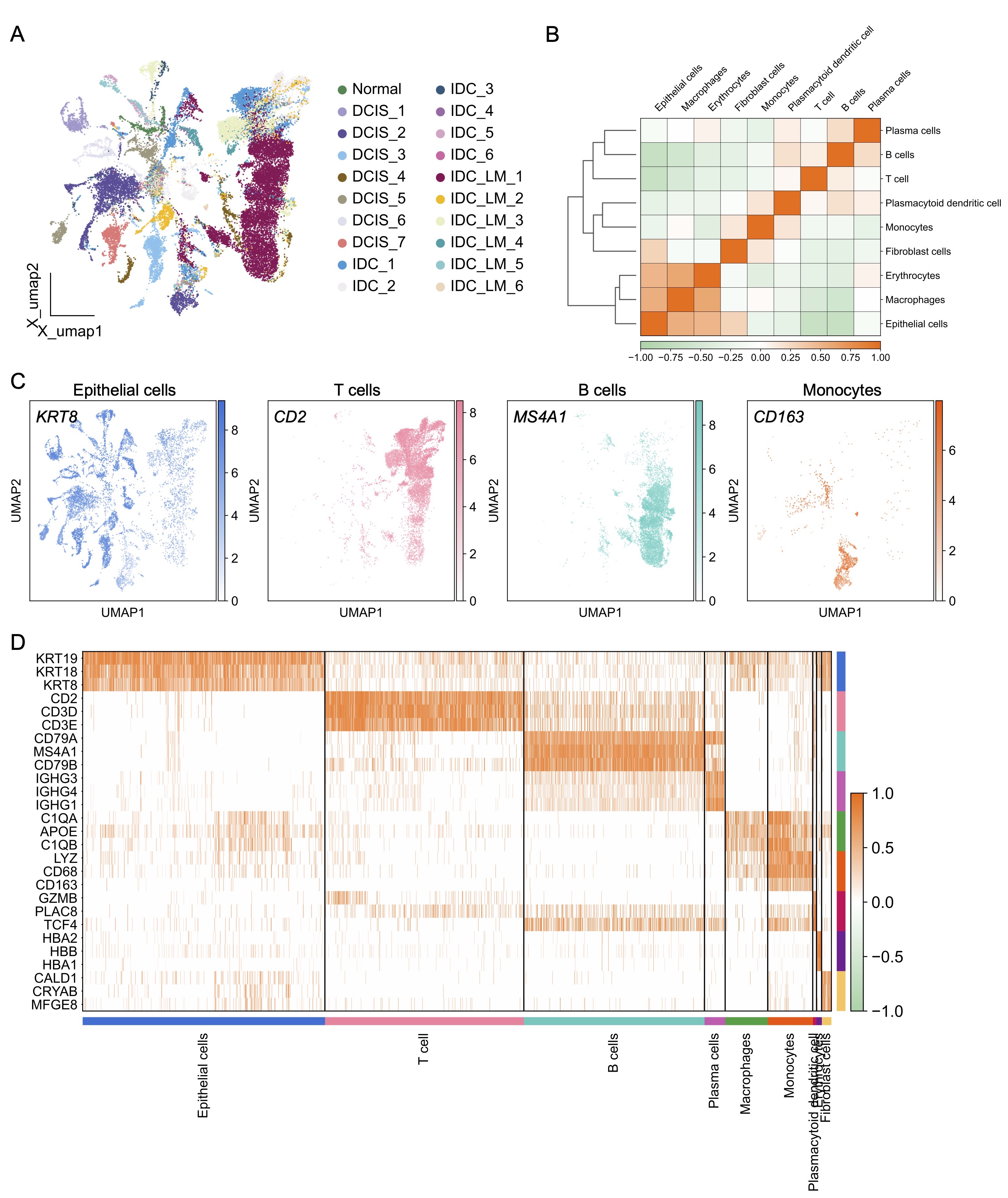

Supplement: Supplementary file 2 — Figure S1 [file 41420_2025_2422_MOESM2_ESM.jpg]

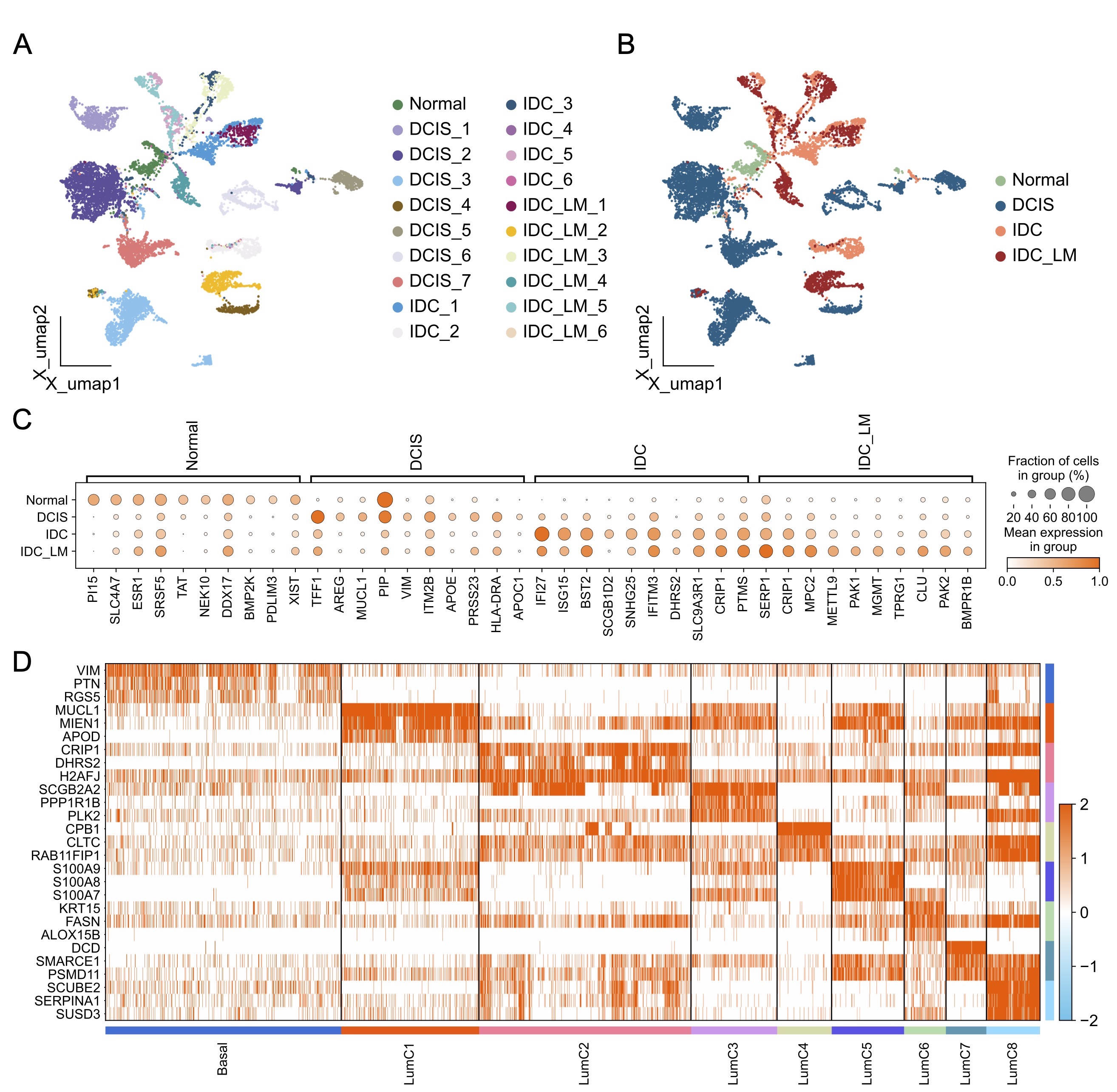

Supplement: Supplementary file 3 — Figure S2 [file 41420_2025_2422_MOESM3_ESM.jpg]

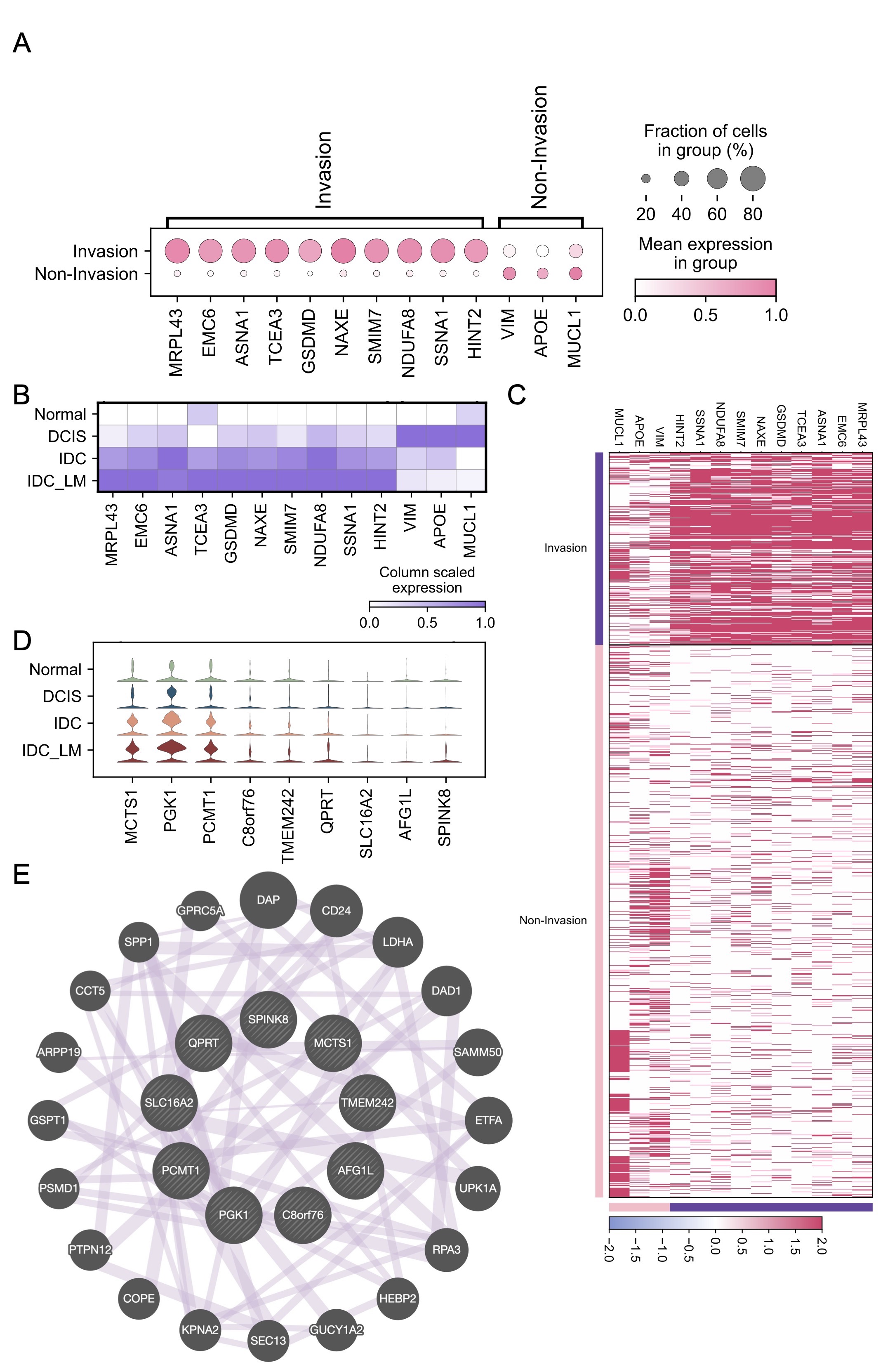

Supplement: Supplementary file 4 — Figure S3 [file 41420_2025_2422_MOESM4_ESM.jpg]

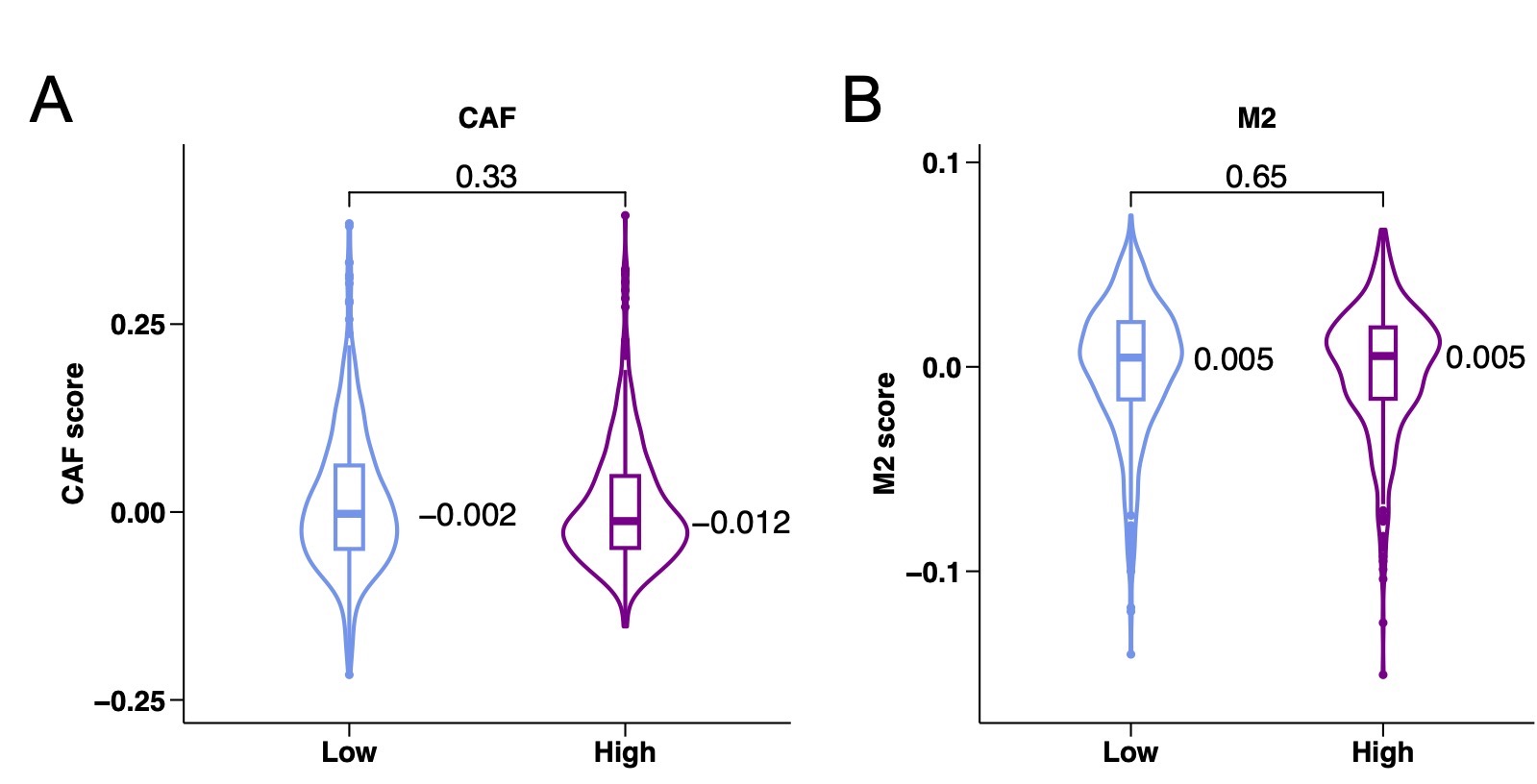

Supplement: Supplementary file 5 — Figure S4 [file 41420_2025_2422_MOESM5_ESM.jpg]

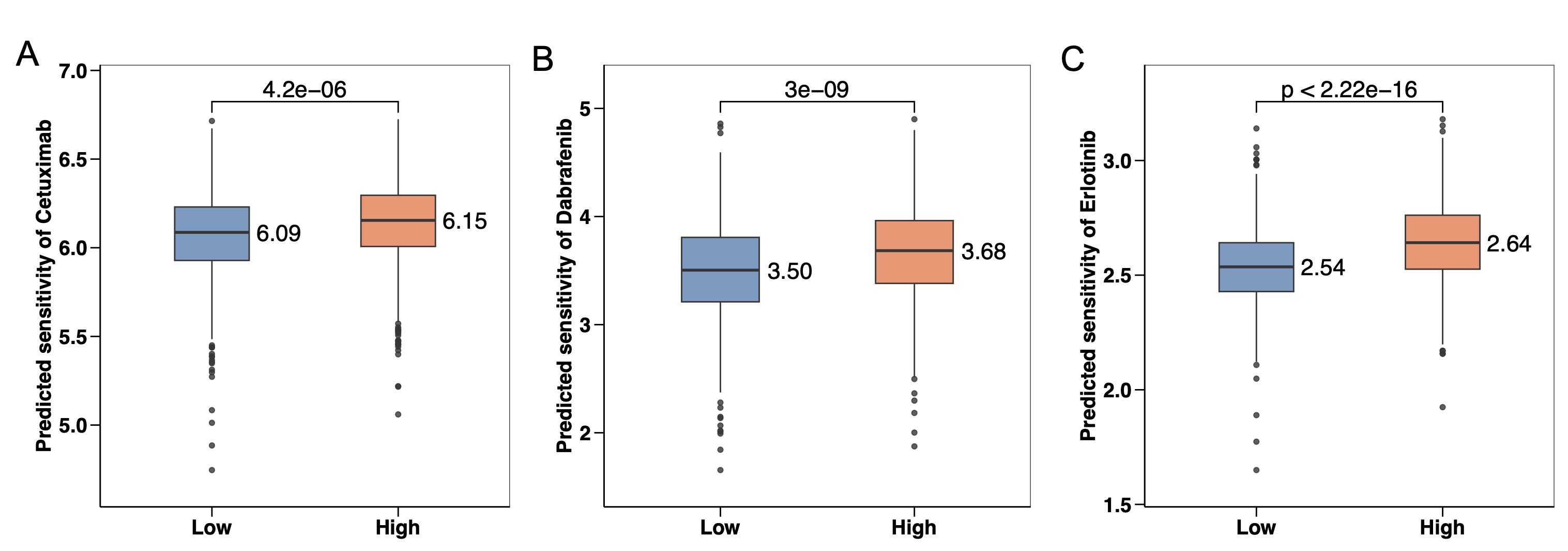

Supplement: Supplementary file 6 — Figure S5 [file 41420_2025_2422_MOESM6_ESM.jpg]
