# Supplementary material for: Development of a breast cancer invasion score to predict tumor aggressiveness and prognosis via PI3K/AKT/mTOR pathway analysis
Source: Cell Death Discov. 2025 Apr 9;11:157. doi: 10.1038/s41420-025-02422-y (PMC11982538; doi:10.1038/s41420-025-02422-y)

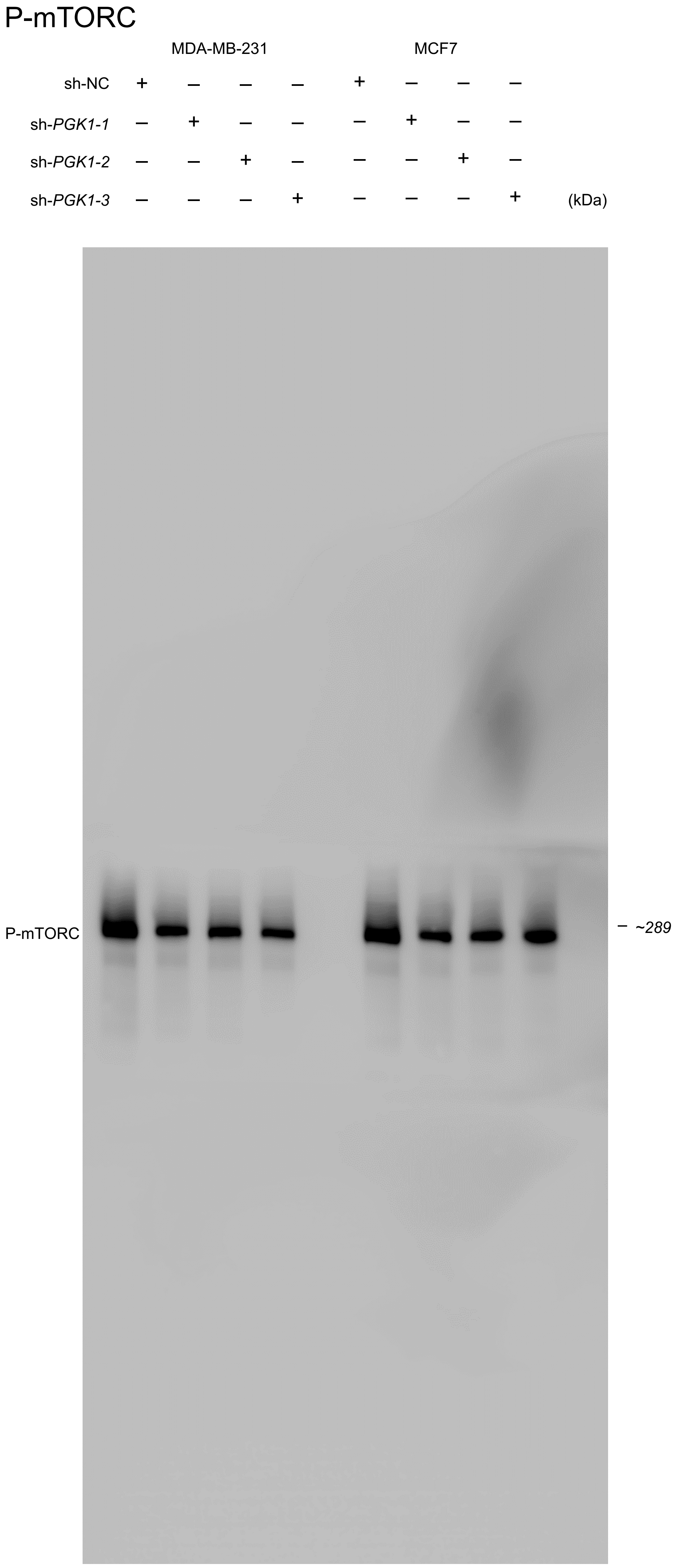
P-mTORC
MDA-MB-231
MCF7
sh-NC + – – – + – – –
sh-*PGK1-1* – + – – – + – –
sh-*PGK1-2* – – + – – – + –
sh-*PGK1-3* – – – + – – – +
(kDa)
P-mTORC
~289

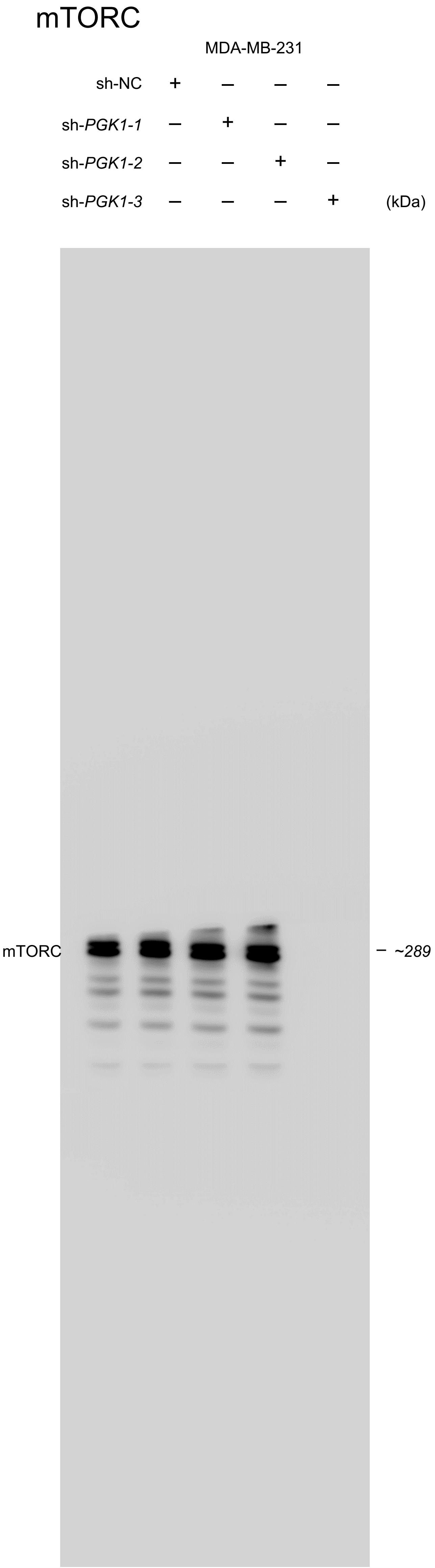
mTORC
MDA-MB-231
sh-NC + – – –
sh-*PGK1-1* – + – –
sh-*PGK1-2* – – + –
sh-*PGK1-3* – – – +
(kDa)
mTORC
~289

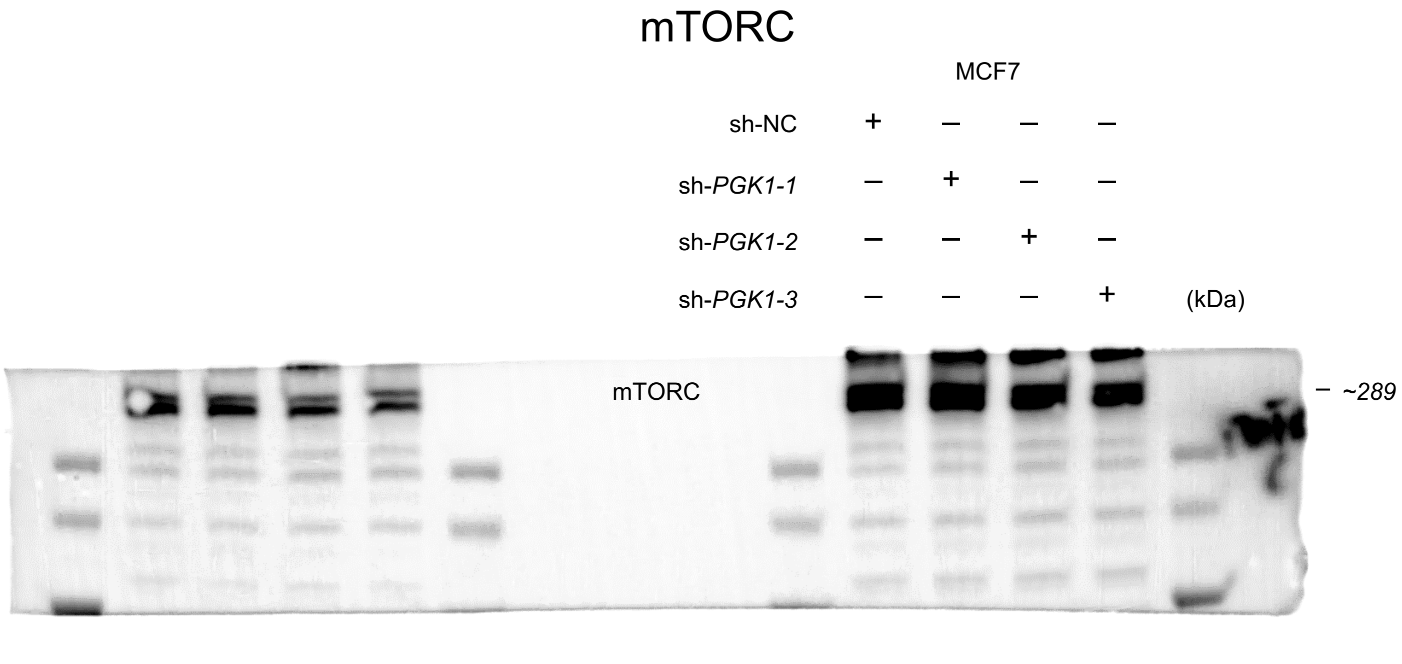
mTORC
MCF7
sh-NC + – – –
sh-*PGK1-1* – + – –
sh-*PGK1-2* – – + –
sh-*PGK1-3* – – – +
(kDa)
mTORC
~289

P-P70S6K

	MDA-MB-231				MCF7				
sh-NC	+	–	–	–	+	–	–	–	
sh-*PGK1-1*	–	+	–	–	–	+	–	–	
sh-*PGK1-2*	–	–	+	–	–	–	+	–	
sh-*PGK1-3*	–	–	–	+	–	–	–	+	(kDa)

P-P70S6K

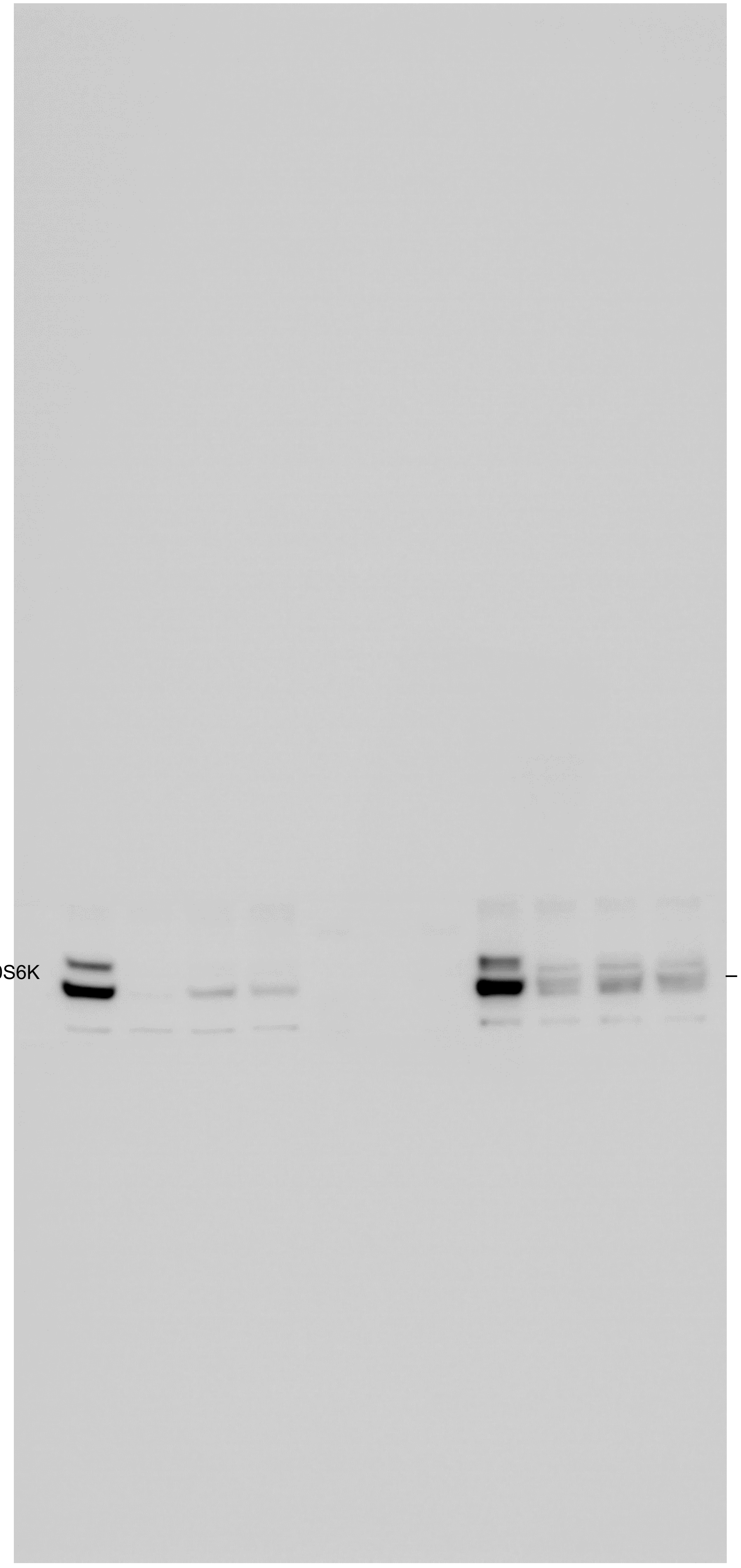

– *~70*

P70S6K

	MDA-MB-231				MCF7				
sh-NC	+	–	–	–	+	–	–	–	
sh-*PGK1-1*	–	+	–	–	–	+	–	–	
sh-*PGK1-2*	–	–	+	–	–	–	+	–	
sh-*PGK1-3*	–	–	–	+	–	–	–	+	(kDa)

P70S6K

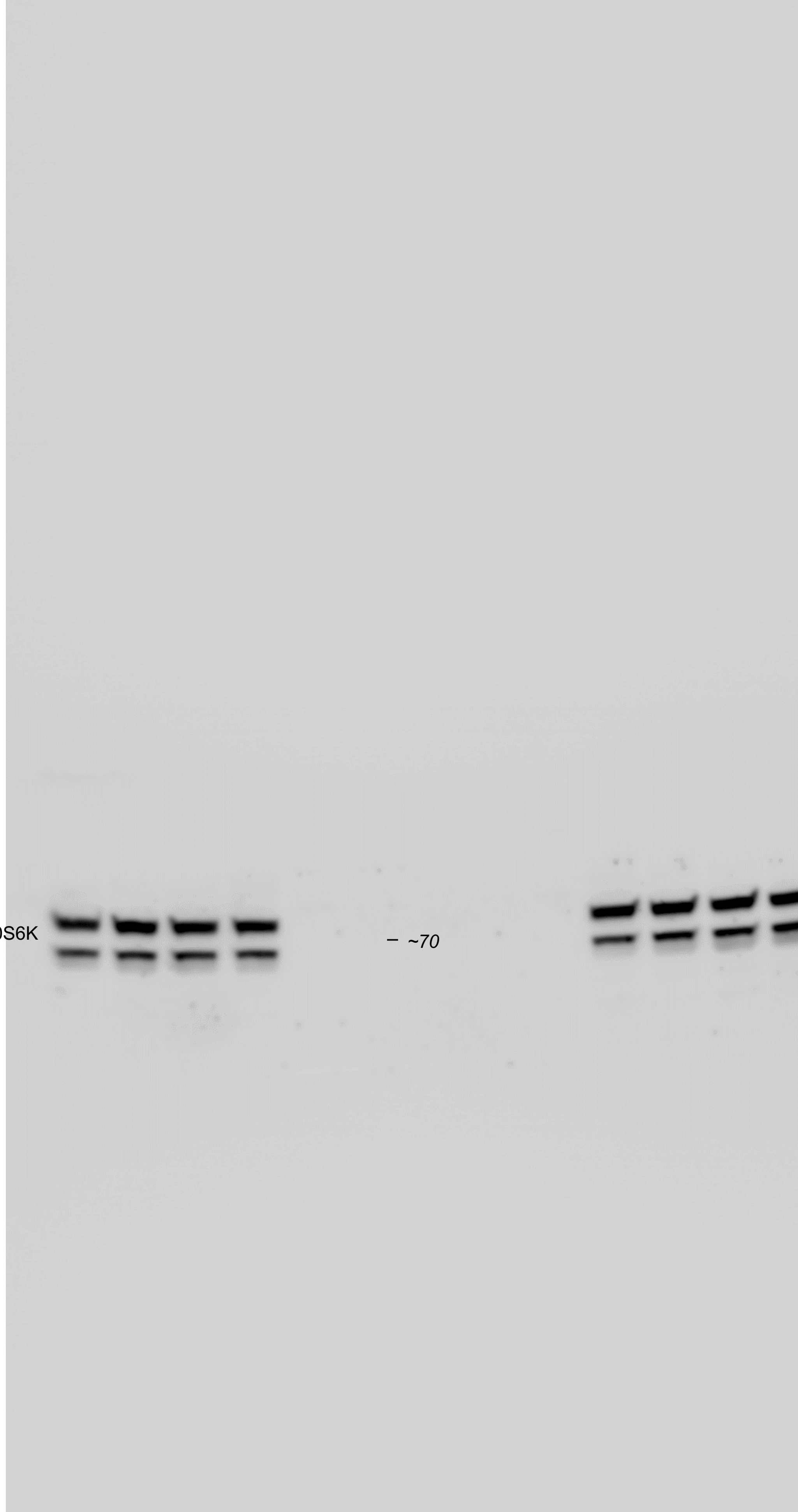

– *~70*

P-S6

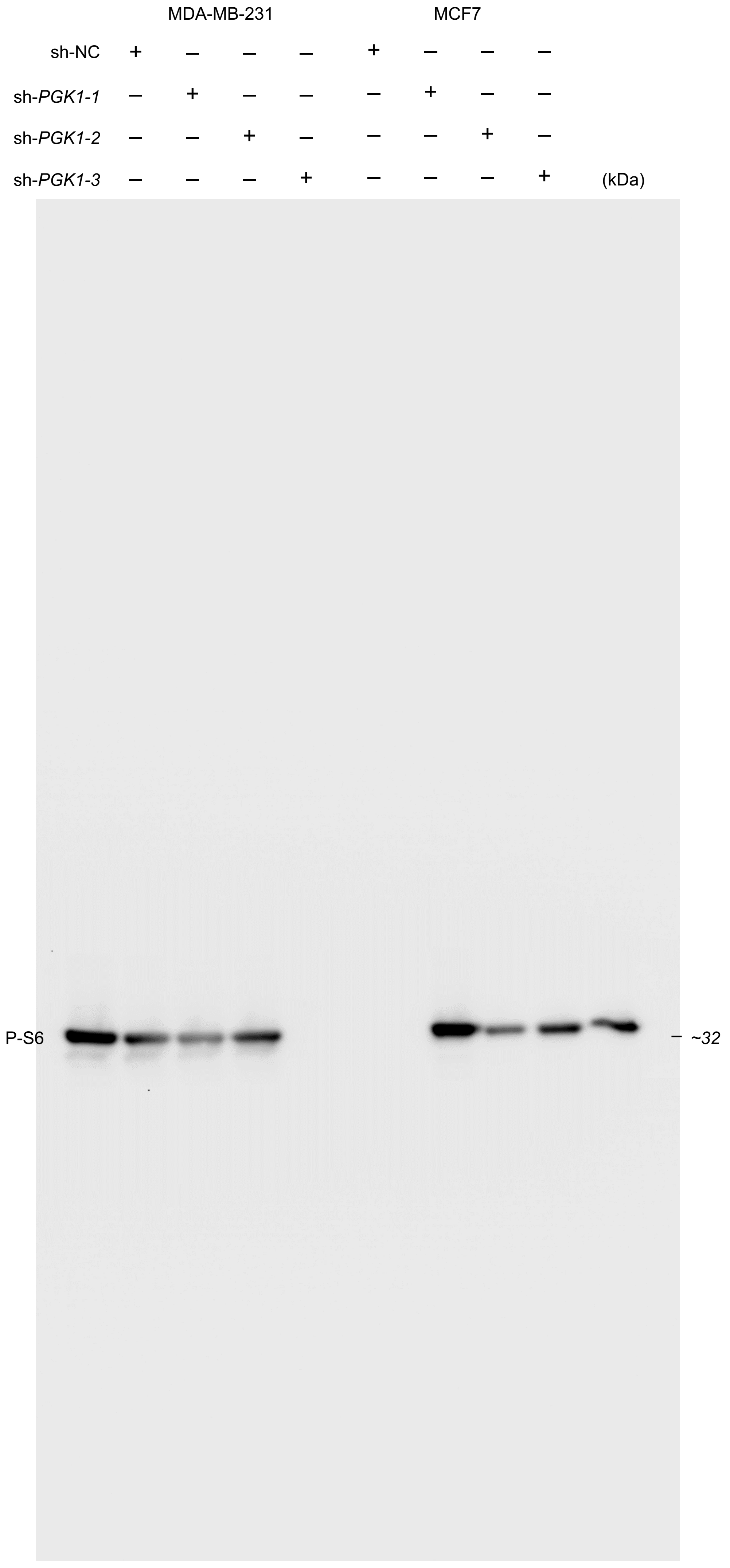
MDA-MB-231
MCF7
sh-NC + – – – + – – –
sh-*PGK1-1* – + – – – + – –
sh-*PGK1-2* – – + – – – + –
sh-*PGK1-3* – – – + – – – +
(kDa)
P-S6
~32

S6

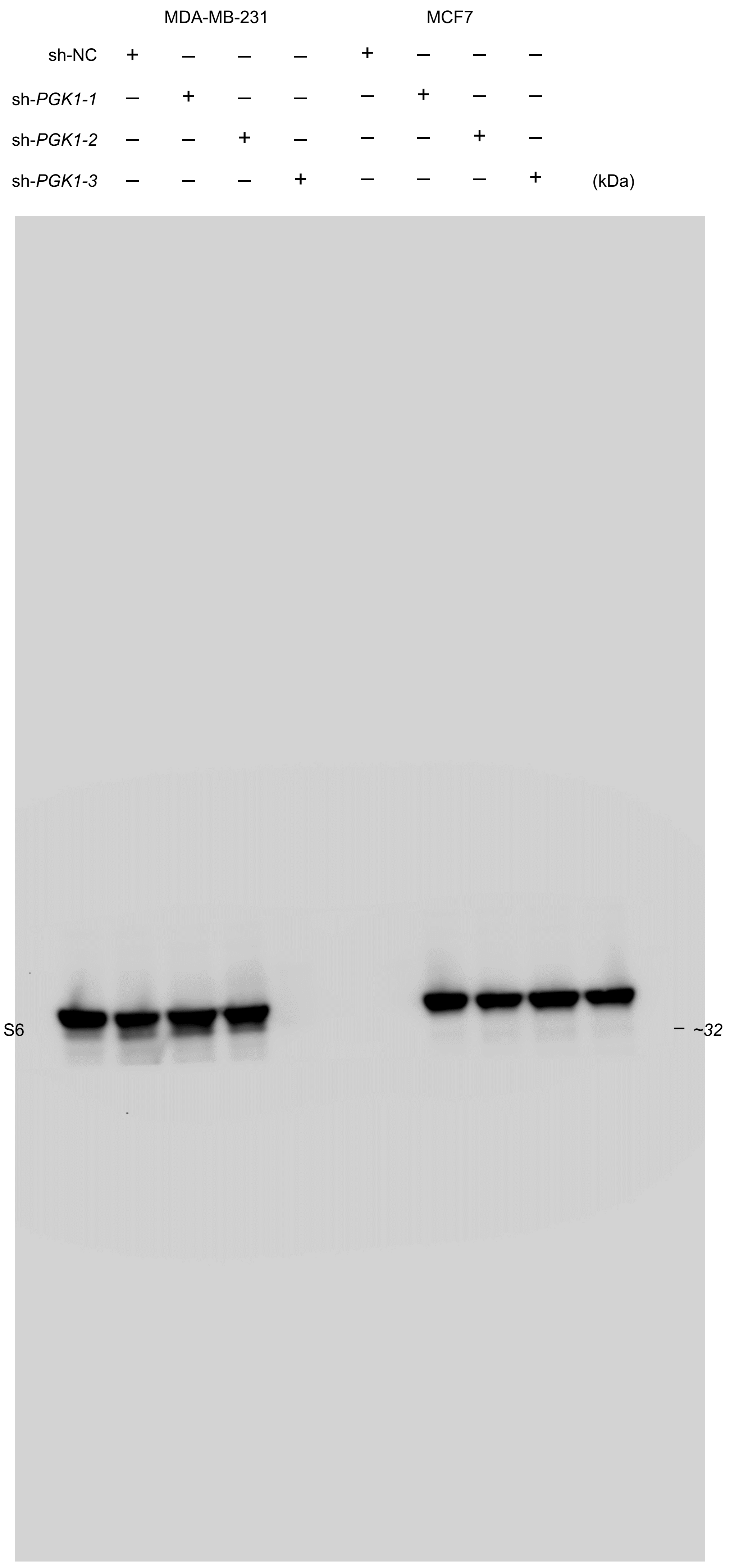
MDA-MB-231
MCF7
sh-NC + – – – + – – –
sh-*PGK1-1* – + – – – + – –
sh-*PGK1-2* – – + – – – + –
sh-*PGK1-3* – – – + – – – +
(kDa)
S6
~32

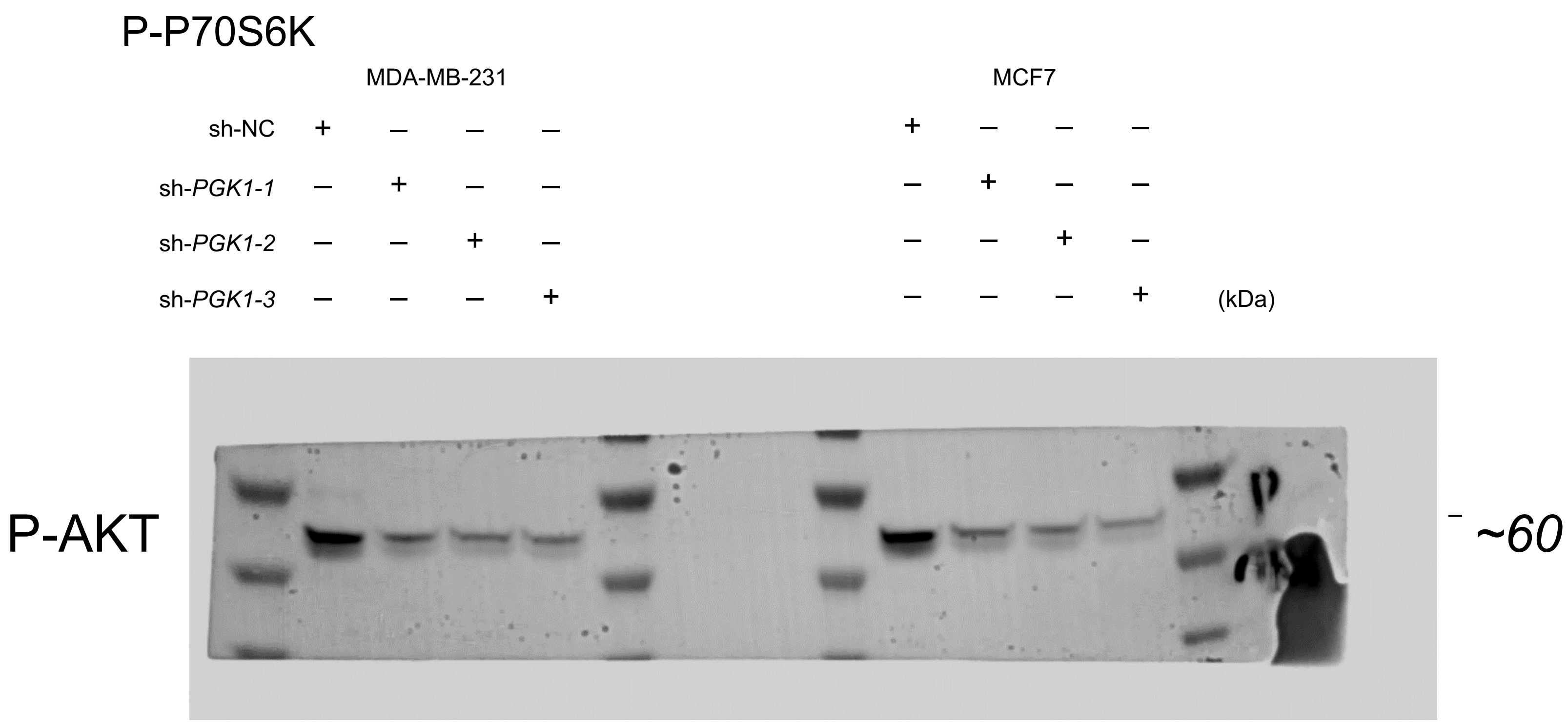
P-P70S6K
MDA-MB-231
MCF7
sh-NC
sh-PGK1-1
sh-PGK1-2
sh-PGK1-3
(kDa)
P-AKT
~60

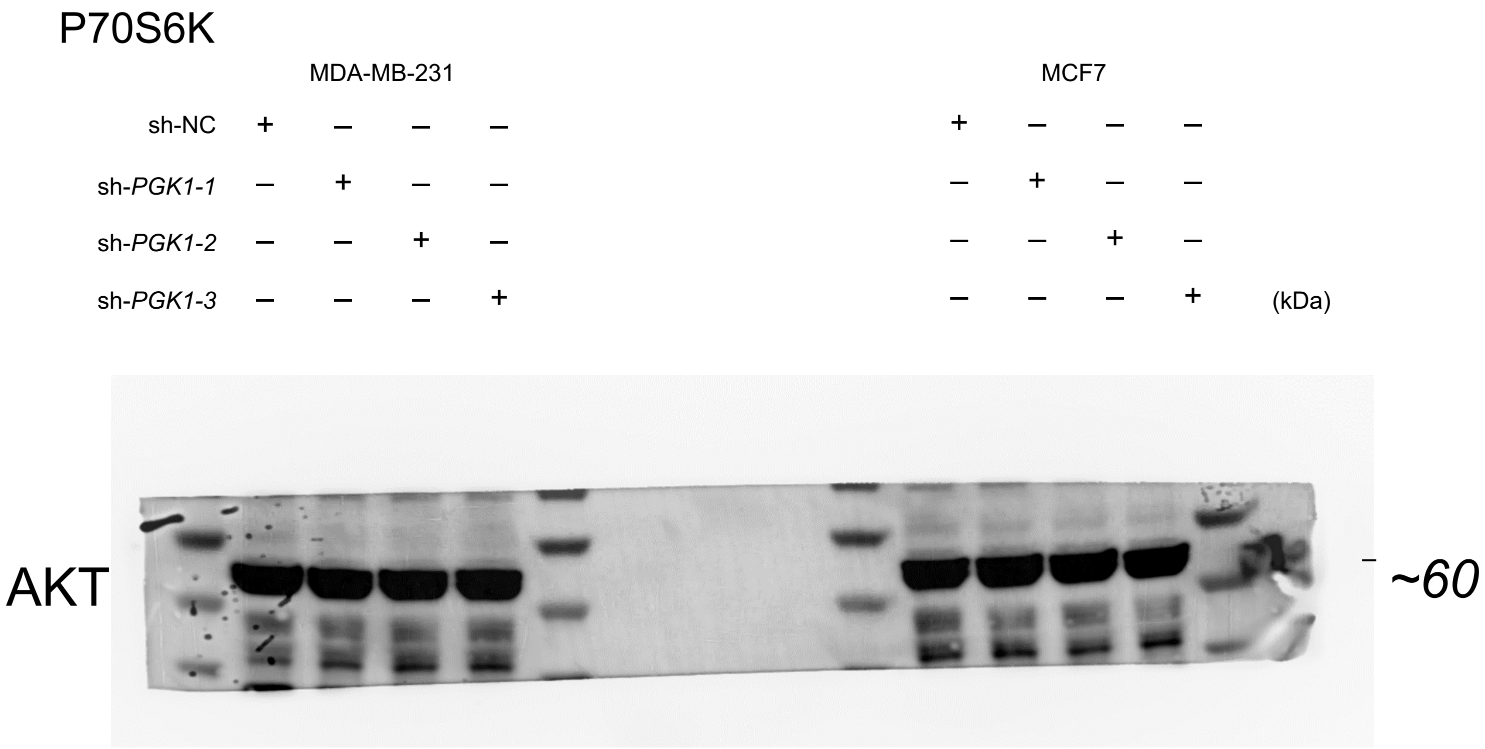
P70S6K
MDA-MB-231
MCF7
sh-NC
sh-PGK1-1
sh-PGK1-2
sh-PGK1-3
(kDa)
AKT
~60

β-actin

	MDA-MB-231				MCF7				
sh-NC	+	–	–	–	+	–	–	–	
sh-*PGK1-1*	–	+	–	–	–	+	–	–	
sh-*PGK1-2*	–	–	+	–	–	–	+	–	
sh-*PGK1-3*	–	–	–	+	–	–	–	+	(kDa)

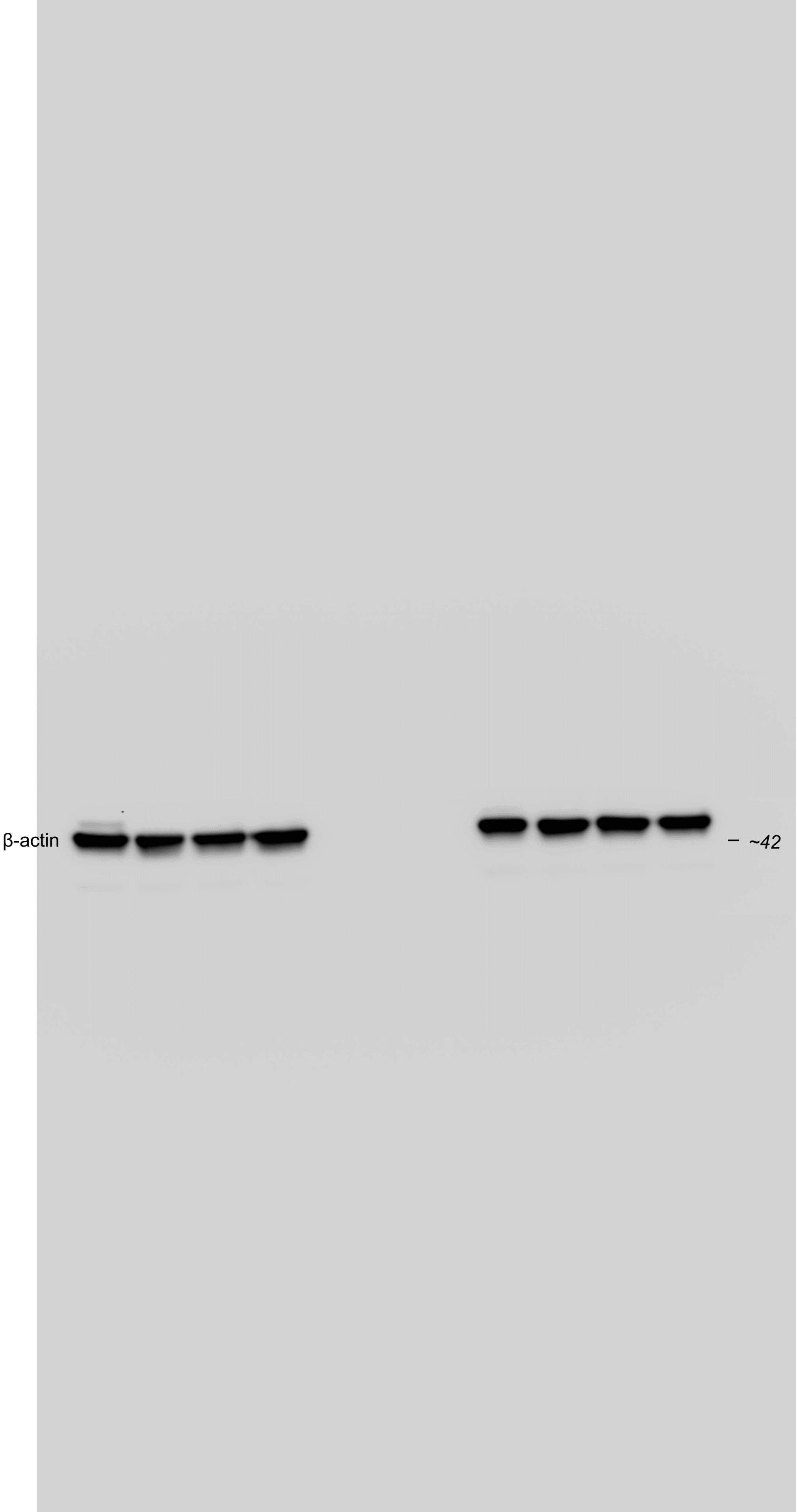

β-actin

– *~42*

P70S6K

	MDA-MB-231				MCF7				
sh-NC	+	–	–	–	+	–	–	–	
sh-*PGK1-1*	–	+	–	–	–	+	–	–	
sh-*PGK1-2*	–	–	+	–	–	–	+	–	
sh-*PGK1-3*	–	–	–	+	–	–	–	+	(kDa)

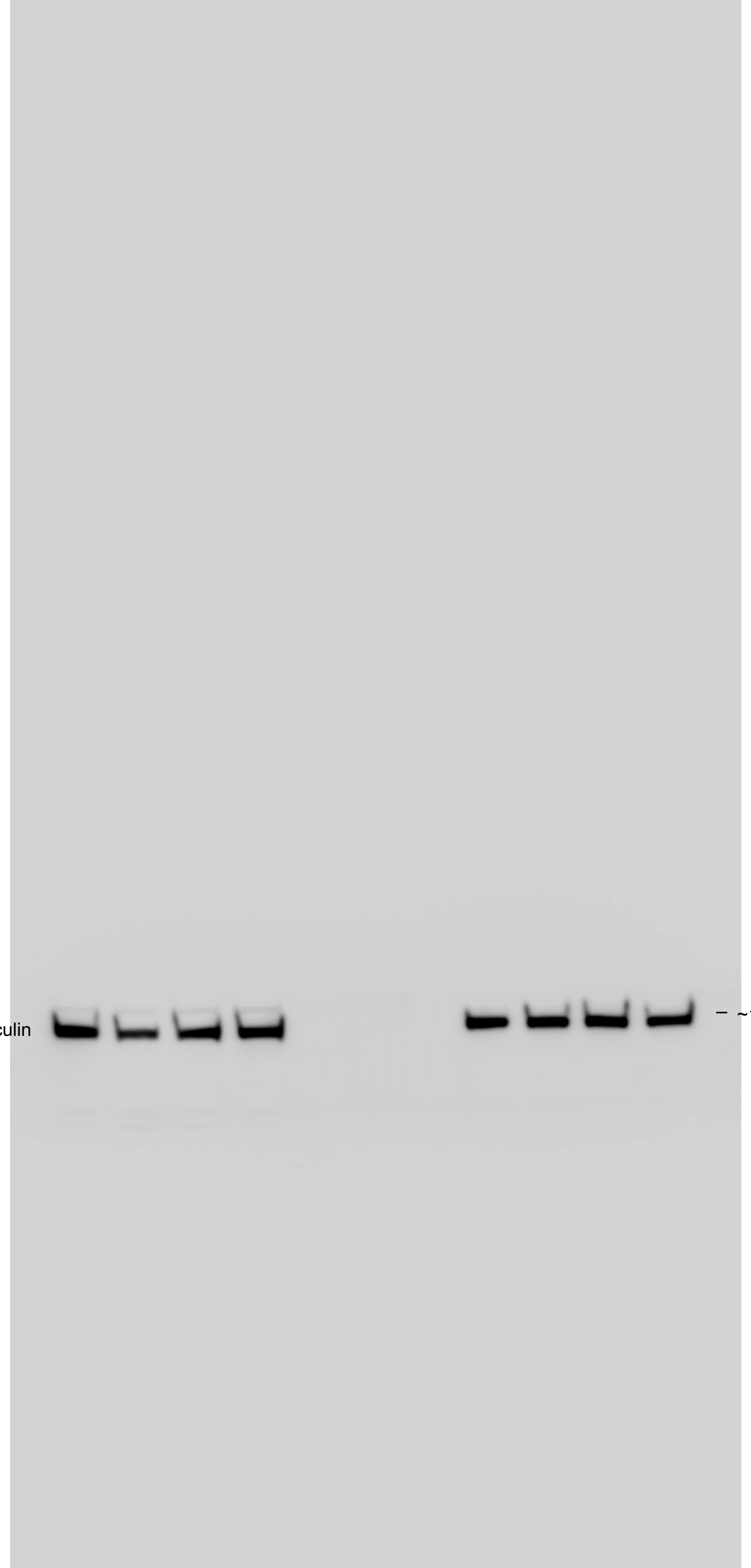

Vinculin

– *~130*

P-mTORC

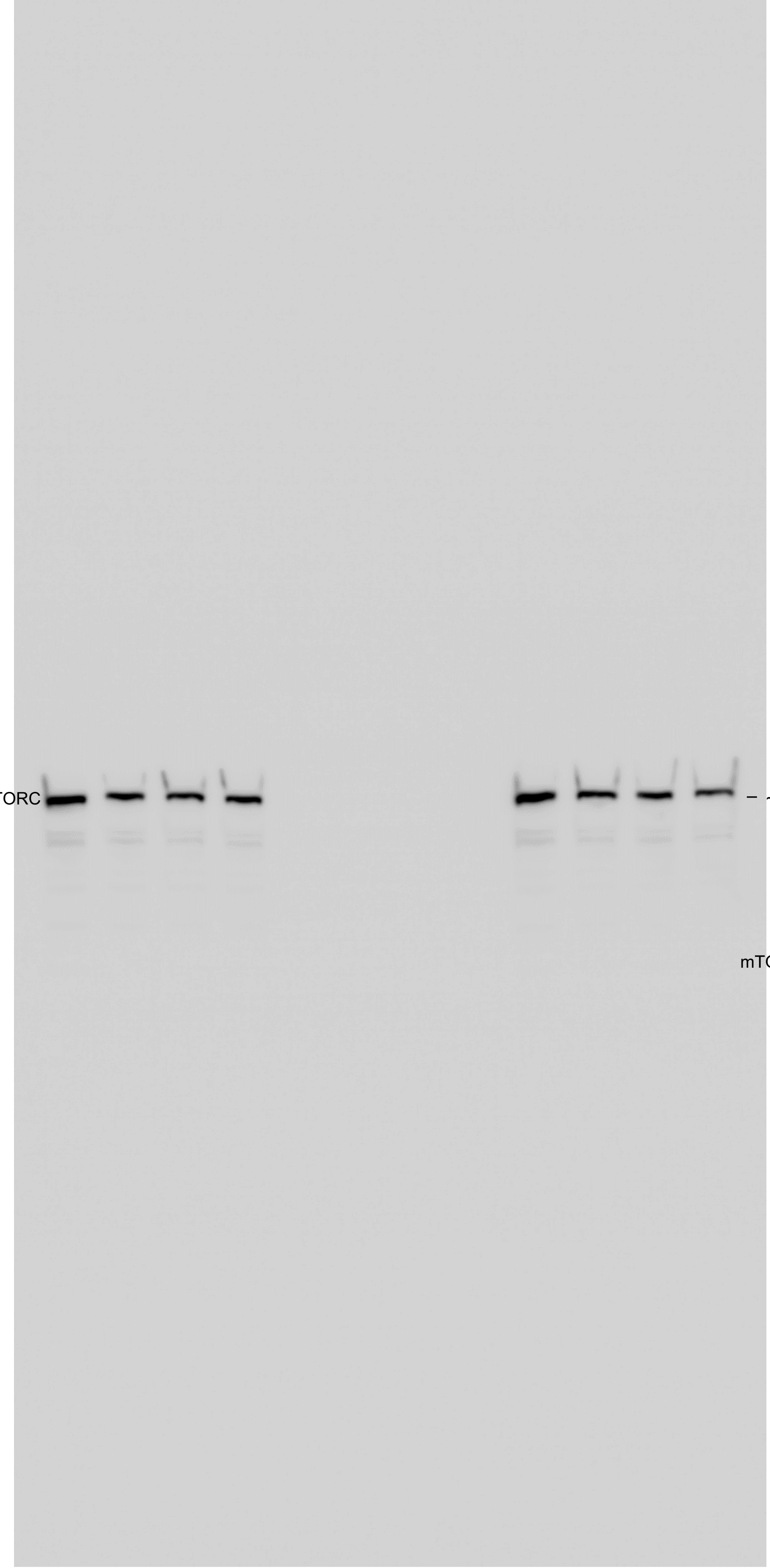

mTORC

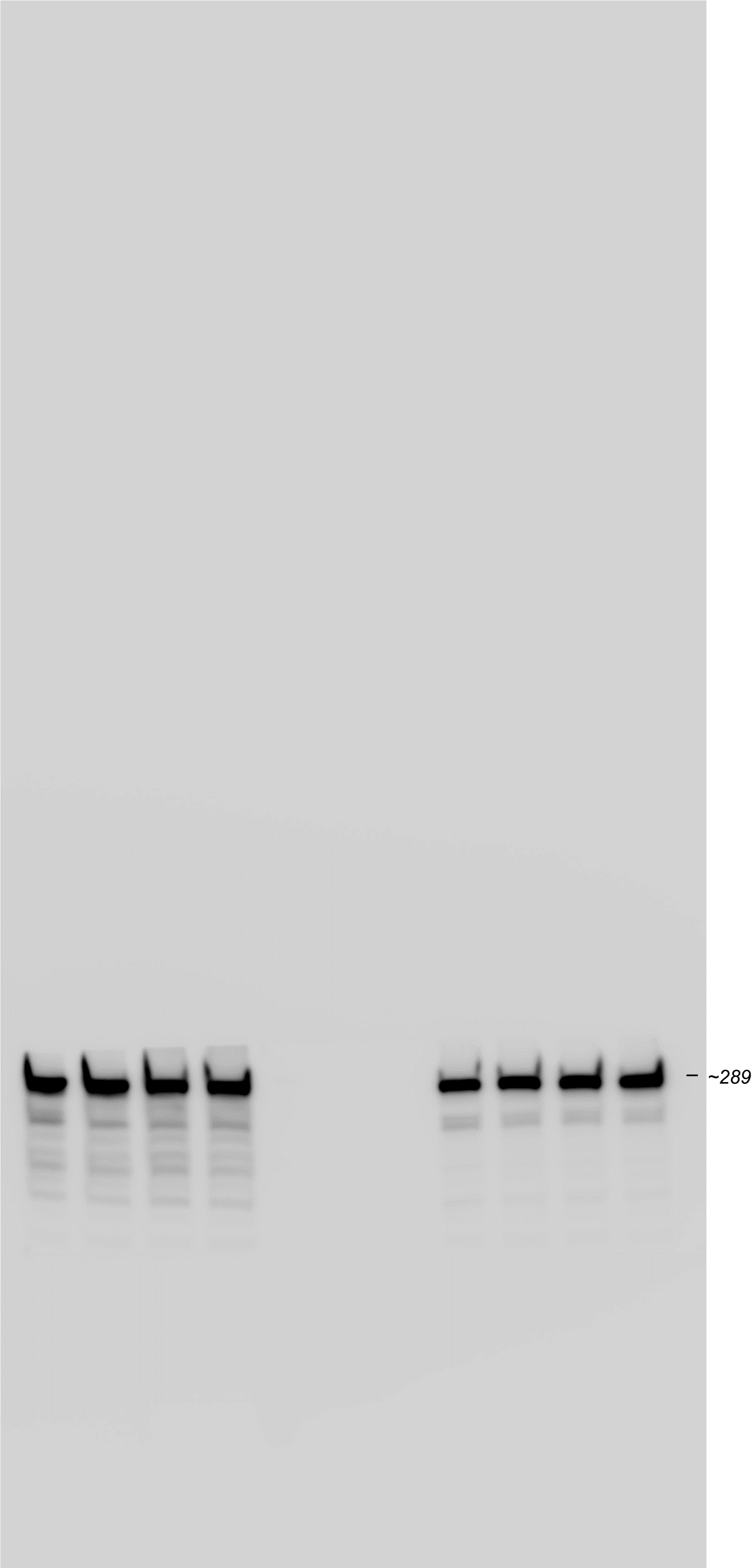

P-P70S6K

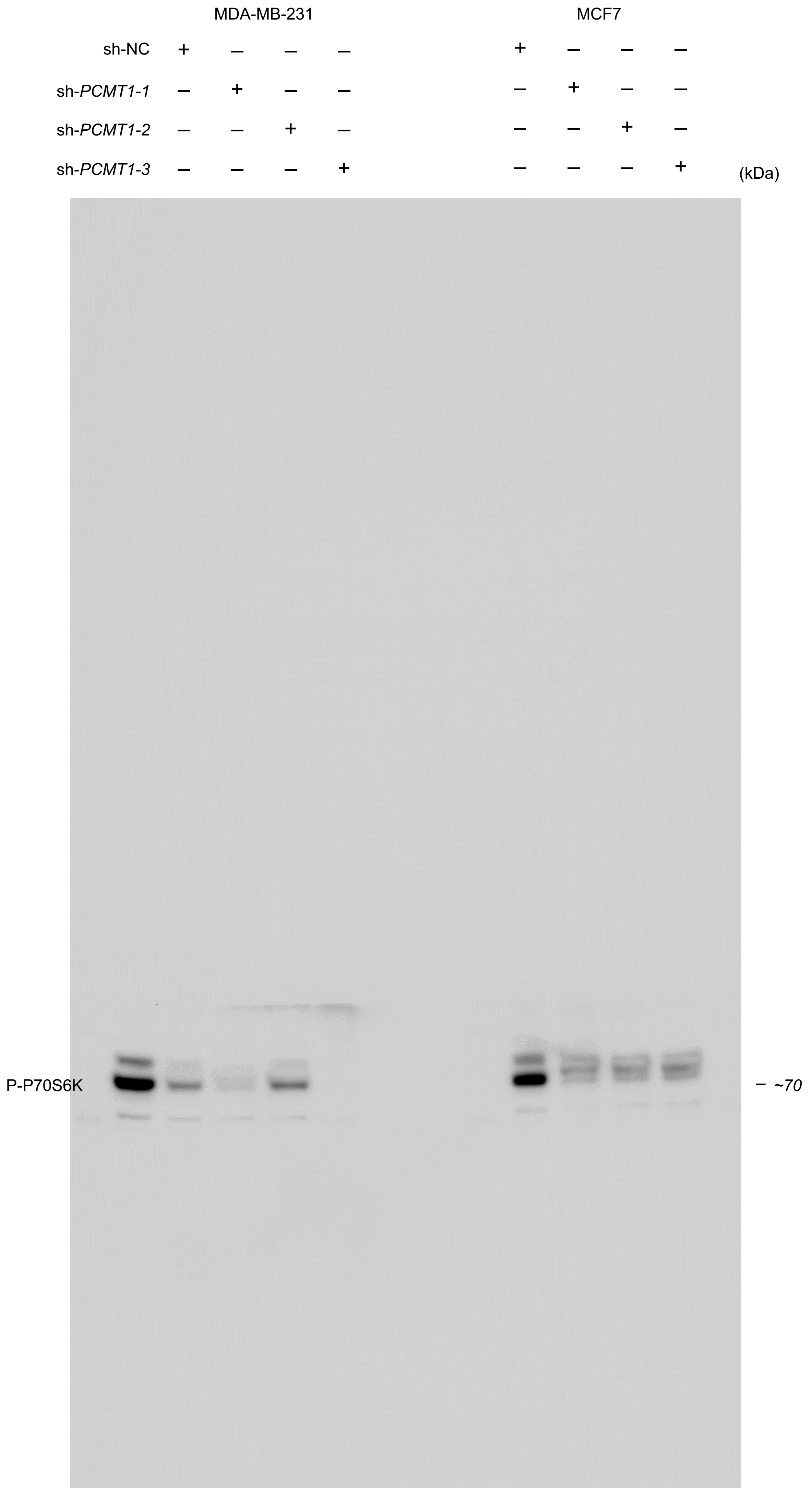

P70S6K

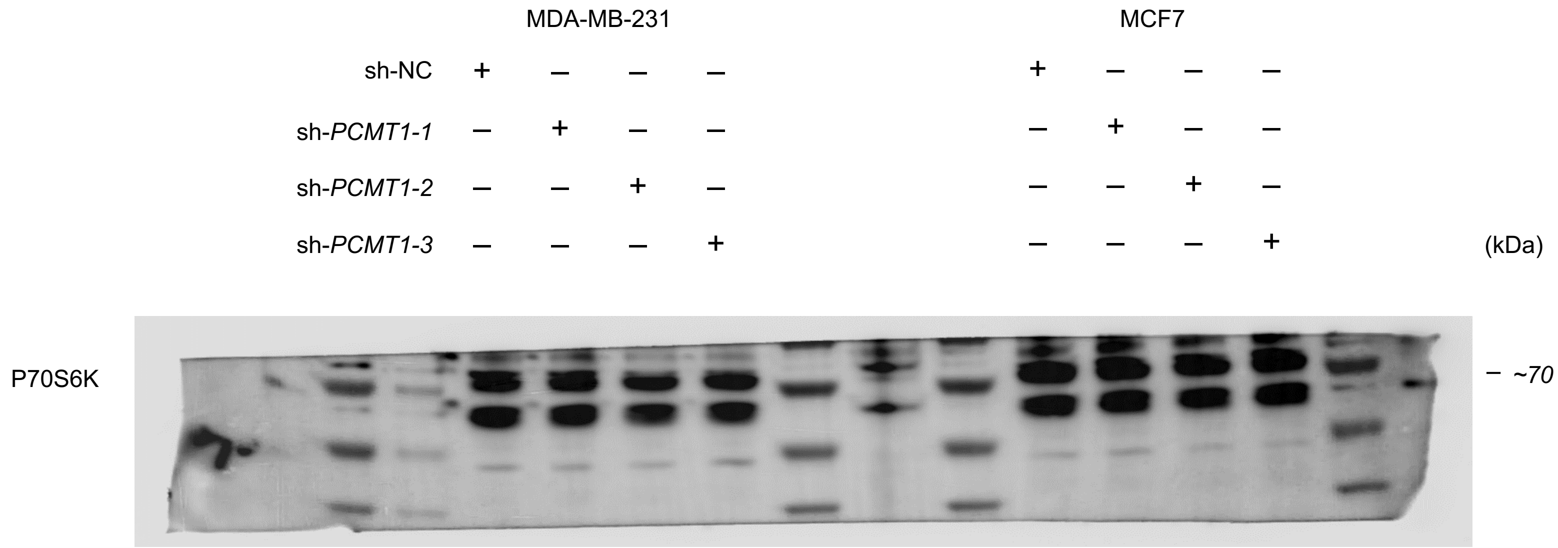

P-S6

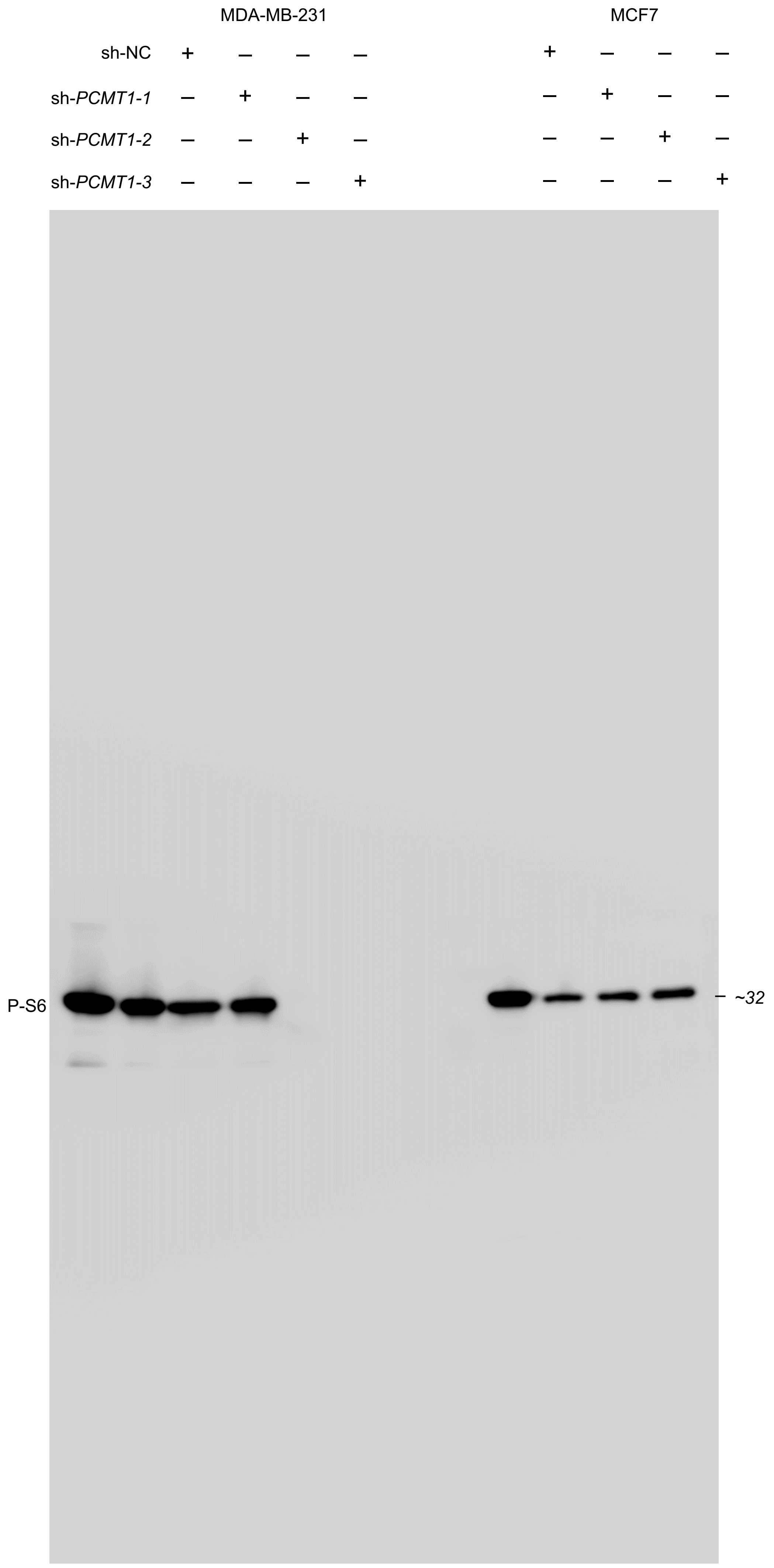
MDA-MB-231
MCF7
sh-NC
sh-PCMT1-1
sh-PCMT1-2
sh-PCMT1-3
(kDa)
P-S6
~32

S6

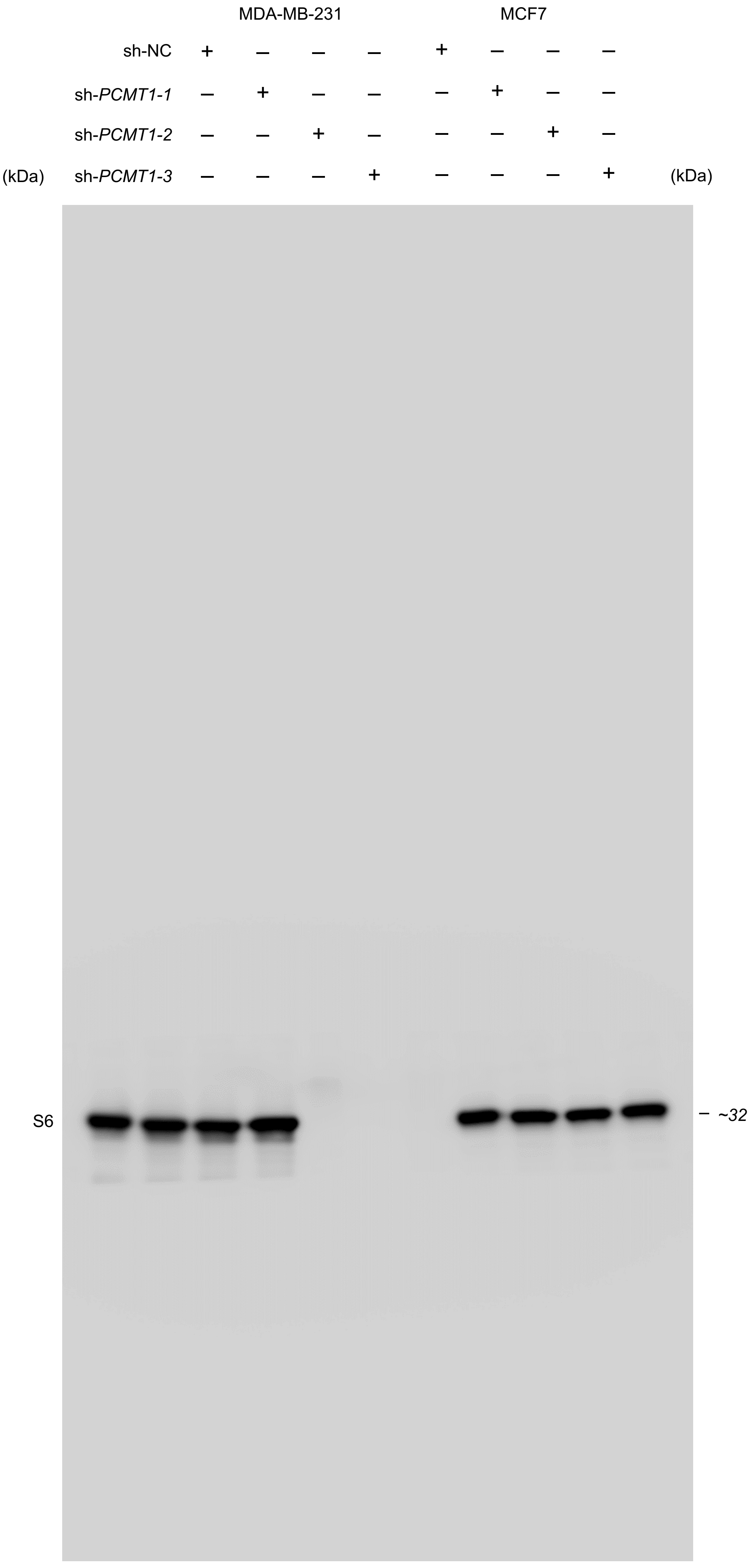
MDA-MB-231
MCF7
sh-NC
sh-PCMT1-1
sh-PCMT1-2
sh-PCMT1-3
(kDa)
S6
~32

P-AKT

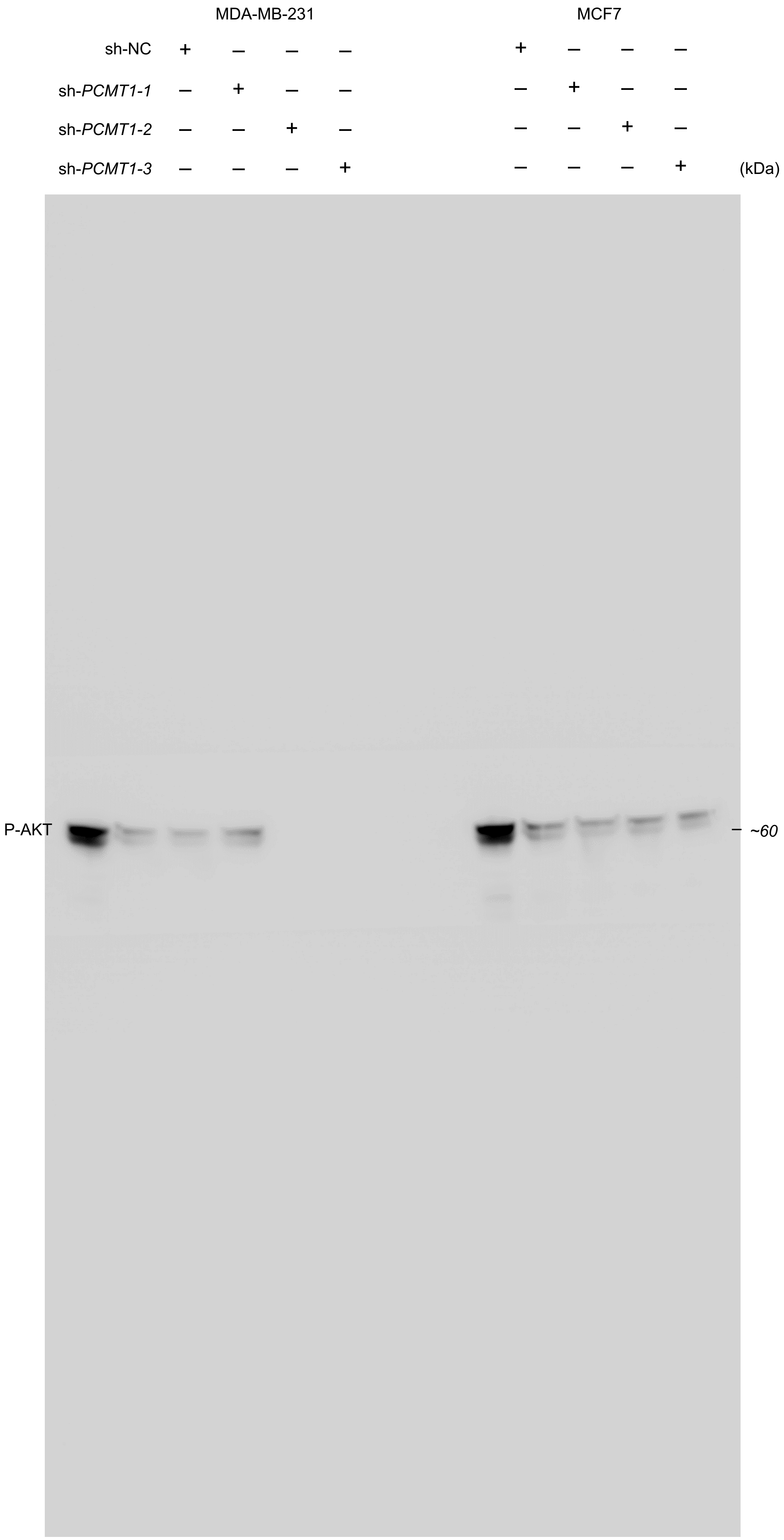
MDA-MB-231
MCF7
sh-NC + – – – + – – –
sh-*PCMT1-1* – + – – – + – –
sh-*PCMT1-2* – – + – – – + –
sh-*PCMT1-3* – – – + – – – +
(kDa)
P-AKT
~60

AKT

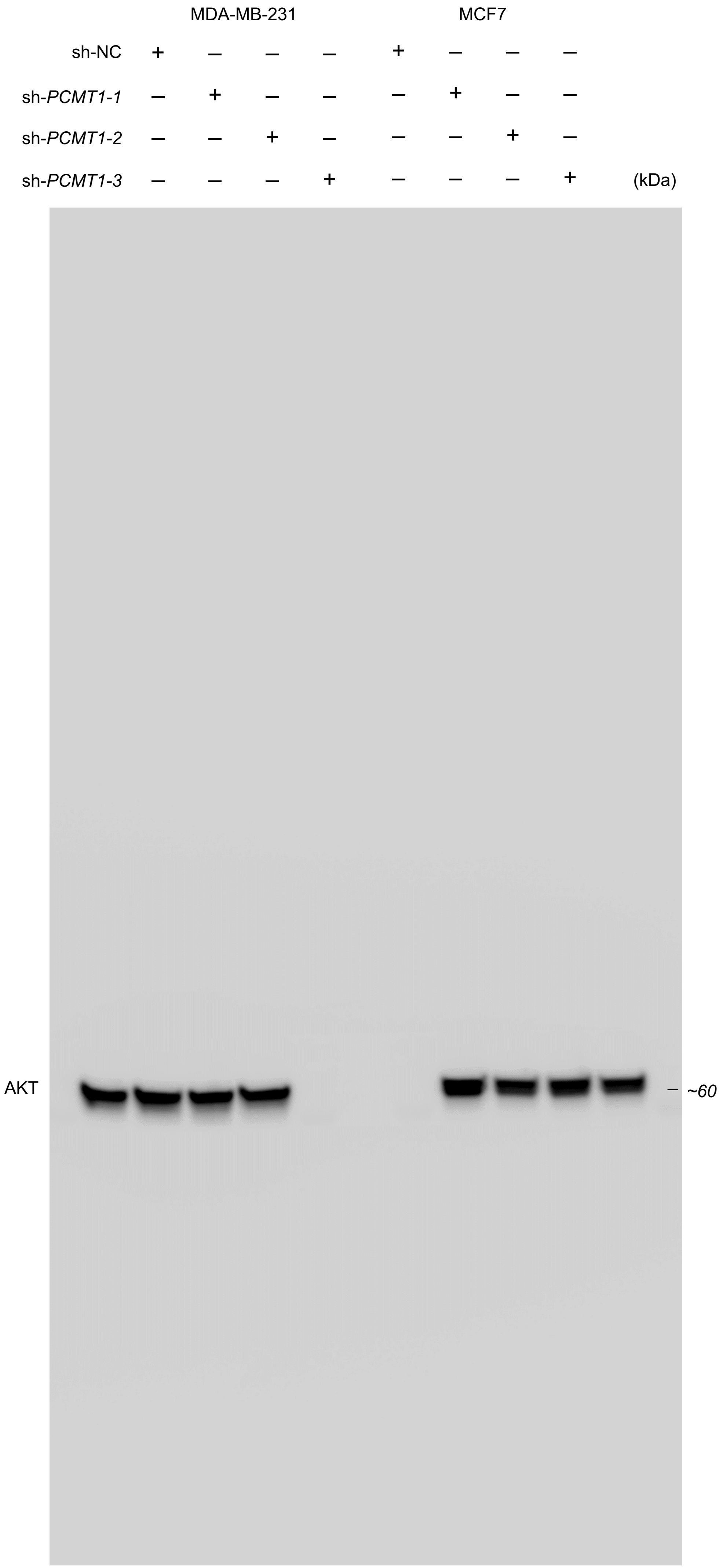
MDA-MB-231
MCF7
sh-NC + – – – + – – –
sh-*PCMT1-1* – + – – – + – –
sh-*PCMT1-2* – – + – – – + –
sh-*PCMT1-3* – – – + – – – +
(kDa)
AKT
~60

β-actin

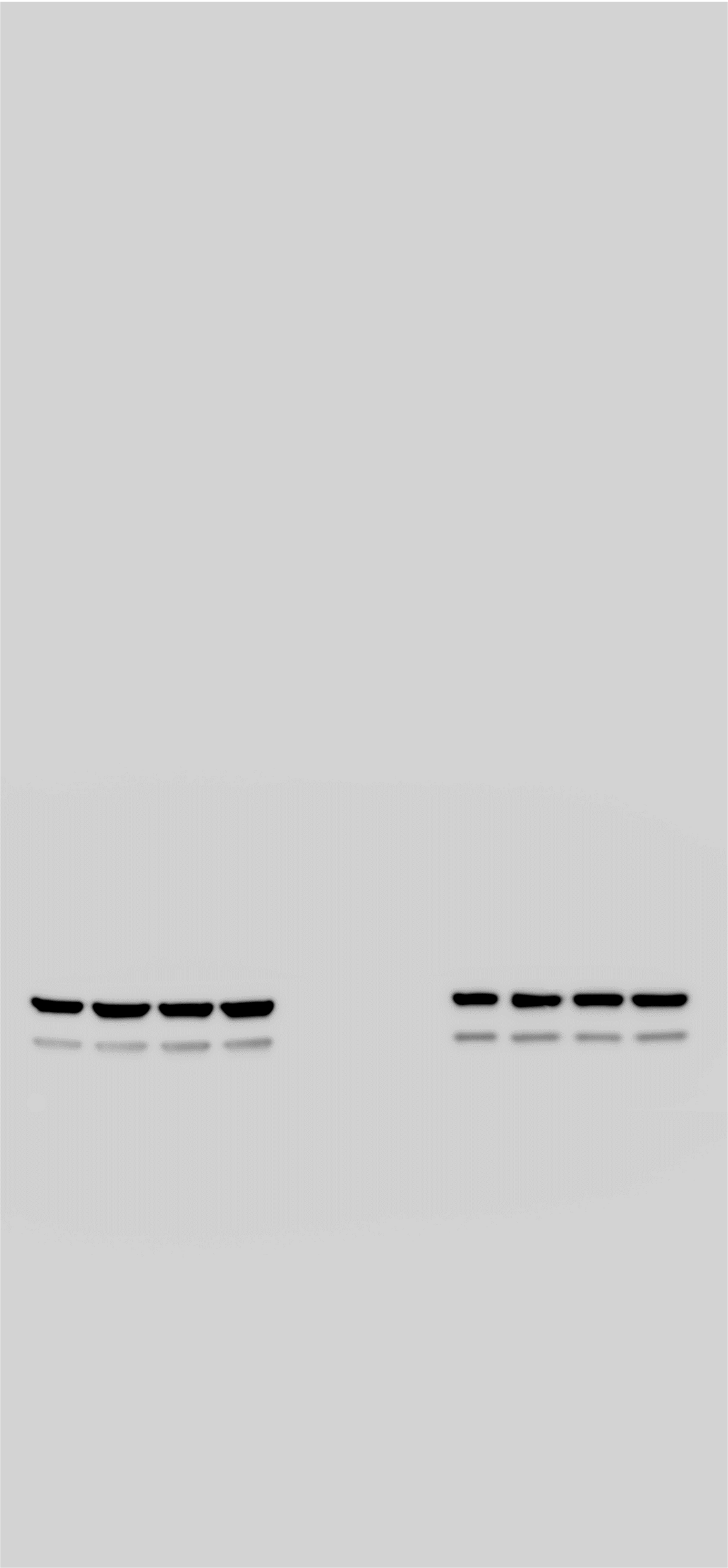

Vinculin

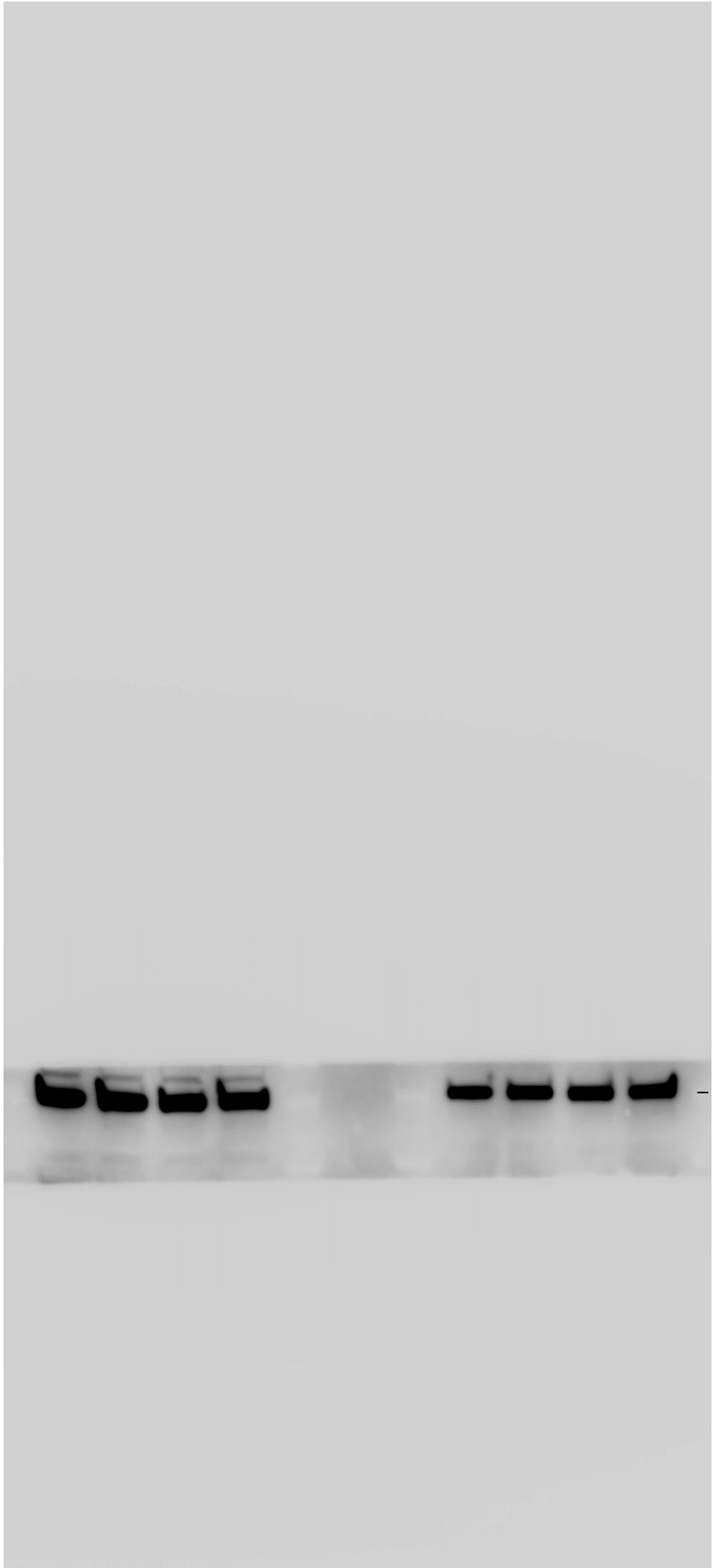

Supplement: Supplementary file 8 — Western Blot [file 41420_2025_2422_MOESM8_ESM.pdf]
